# Quantitative proteomic comparison of salt stress in *Chlamydomonas reinhardtii* and the snow alga *Chlamydomonas nivalis* reveals mechanisms for salt-triggered fatty acid accumulation via reallocation of carbon resources

**DOI:** 10.1186/s13068-021-01970-6

**Published:** 2021-05-22

**Authors:** E. Hounslow, C. A. Evans, J. Pandhal, T. Sydney, N. Couto, T. K. Pham, D. James Gilmour, P. C. Wright

**Affiliations:** 1 Department of Chemical and Biological Engineering, University of Sheffield, Mappin Street, Sheffield, S1 3JD UK; 2 Department of Chemistry, University of Sheffield, Sheffield, S3 7HF UK; 3 Department of Molecular Biology and Biotechnology, Firth Court, University of Sheffield, Western Bank, Sheffield, S10 2TN UK; 4 University of Southampton, University Road, Southampton, SO17 1BJ UK

**Keywords:** Quantitative proteomics, *Chlamydomonas nivalis*, *Chlamydomonas reinhardtii*, Fatty acid production, Salt stress, Biofuel

## Abstract

**Background:**

*Chlamydomonas reinhardtii* is a model green alga strain for molecular studies; its fully sequenced genome has enabled omic-based analyses that have been applied to better understand its metabolic responses to stress. Here, we characterised physiological and proteomic changes between a low-starch *C. reinhardtii* strain and the snow alga *Chlamydomonas nivalis,* to reveal insights into their contrasting responses to salinity stress.

**Results:**

Each strain was grown in conditions tailored to their growth requirements to encourage maximal fatty acid (as a proxy measure of lipid) production, with internal controls to allow comparison points. In 0.2 M NaCl, *C. nivalis* accumulates carbohydrates up to 10.4% DCW at 80 h, and fatty acids up to 52.0% dry cell weight (DCW) over 12 days, however, *C. reinhardtii* does not show fatty acid accumulation over time, and shows limited carbohydrate accumulation up to 5.5% DCW. Analysis of the *C. nivalis* fatty acid profiles showed that salt stress improved the biofuel qualities over time. Photosynthesis and respiration rates are reduced in *C. reinhardtii* relative to *C. nivalis* in response to 0.2 M NaCl. De novo sequencing and homology matching was used in conjunction with iTRAQ-based quantitative analysis to identify and relatively quantify proteomic alterations in cells exposed to salt stress. There were abundance differences in proteins associated with stress, photosynthesis, carbohydrate and lipid metabolism proteins. In terms of lipid synthesis, salt stress induced an increase in dihydrolipoyl dehydrogenase in *C. nivalis* (1.1-fold change), whilst levels in *C. reinhardtii* remained unaffected; this enzyme is involved in acetyl CoA production and has been linked to TAG accumulation in microalgae. In salt-stressed *C. nivalis* there were decreases in the abundance of UDP-sulfoquinovose (− 1.77-fold change), which is involved in sulfoquinovosyl diacylglycerol metabolism, and in citrate synthase (− 2.7-fold change), also involved in the TCA cycle. Decreases in these enzymes have been shown to lead to increased TAG production as fatty acid biosynthesis is favoured. Data are available via ProteomeXchange with identifier PXD018148.

**Conclusions:**

These differences in protein abundance have given greater understanding of the mechanism by which salt stress promotes fatty acid accumulation in the un-sequenced microalga *C. nivalis* as it switches to a non-growth state, whereas *C. reinhardtii* does not have this response.

**Supplementary Information:**

The online version contains supplementary material available at 10.1186/s13068-021-01970-6.

## Background

Microalgae can divert cellular resources towards lipid accumulation under conditions of stress, and identifying the molecular trigger for this response could aid in engineering improved lipid productivity, a precursor for biofuel production. Nitrogen deprivation is typically used as the stressor or "lipid trigger". The majority of molecular research in this area has focused on *Chlamydomonas reinhardtii*. This microalga has a full genome sequence available and represents a well-characterised model green alga [1].

High salinity is an overlooked stressor for lipid accumulation, largely due to the relatively low number of microalgae that have been identified to respond in this manner. However, proteomics has been applied to study salt stress in a variety of photosynthetic systems [2, 3]. Increased salinity has been shown to enhance lipid content in *Dunaliella salina* [4] and mixed microalgal cultures [5], and to increase triacylglycerol (TAG) levels in *Nannochloropsis salina* [6]. High NaCl has also been shown to increase lipid productivity in the freshwater algal species *Desmodesmus abundans* [7]. Moreover, the response can include changes in fatty acid profiles, for example, *Botryococcus braunii* shows increases in the proportions of oleic acid (from 13.35 to 28.28% of total lipid) and palmitic acid (from 15.14 to 33.76% of total lipid) under increased salinity [8]. The effect of 100 mM NaCl salt stress on lipid content in wild-type CC124 *C. reinhardtii* has revealed a 50-fold increase from approximately 0.1 µg TAG 10^–6^ cells to approximately 5 µg TAG 10^–6^ cells [9], to reach similar levels to those found for this strain under nitrogen deprivation. Our previous work also investigated salt stress and lipid accumulation in a low-starch mutant *C. reinhardtii* strain (Ball I7, CC 4325), which also shows enhanced lipid accumulation capabilities under nitrogen deprivation [10]. We found that 100 mM NaCl induced small but significant increases in the fatty acid methyl ester (FAME) content over time (8.23% of dry cell weight (DCW) compared with 6.21% in control conditions) [11]. FAME measurements are used to quantify and investigate the lipid profile of a sample, and are especially useful for assessing biodiesel potential. Although increases in FAME content were found under higher NaCl concentrations (300 mM) in the short term (0 h), long-term (up to 76 h) FAME reduced substantially as the cells were eventually killed by these salinity levels. The relatively small increases observed in both previous studies suggest that although salt can affect the physiology of the low-starch mutant of *C. reinhardtii* cells, it does not have a significant fatty acid accumulation response.

As proteins are the functional entities in cells, proteomics is a proven tool to analyse metabolic responses to stressors such as nutrient deprivation, and has previously been applied to provide a unique profile of metabolic changes associated with the nitrogen deprivation lipid trigger [12]. There have been several proteomic studies to understand the metabolic consequences of salt stress in *C. reinhardtii* [13, 14], including studies focusing primarily on photosystem protein changes [15, 16]. These studies were directed to responses from photosynthetic proteins, housekeeping proteins and amino acid metabolism (especially proline metabolism), rather than lipid production. One study revealed inhibitory effects of 50–150 mM NaCl on photosystem (PS) II and light harvesting complex (LHC) II functionality and on flagella motility [15]. Similarly, Subramanyam et al. [16] targeted PSI and LHCI proteins from PSI-LHCI supercomplexes in *C. reinhardtii* cells grown under 100 mM NaCl conditions. They found that electron transfer activity decreased, and there is additional evidence that PSI-LHCI is damaged by reactive oxygen species (ROS) under high salt conditions, causing impaired cell growth. Yokthongwattana et al. [13] used 300 mM NaCl with an exposure time of 2 h to investigate the effect of short-term sudden salt stress on the *C. reinhardtii* proteome, and concluded that under salt stress, antioxidant enzymes are required to scavenge the ROS. The cells require significant energy to maintain homeostasis from the glycolytic and energy-producing metabolic pathways, and heat shock and chaperone proteins are required to refold aggregated or mis-folded proteins.

Often referred to as a “snow alga”, *Chlamydomonas nivalis* is a close relative of *C. reinhardtii. C. nivalis* is a primary coloniser in frozen and snowy environments [17], with the ability to grow with limited light. Cell division can take place between 0 AND 2 °C [18], although the optimum temperature for growth has also been reported as 15–20 °C [18]. Lipid productivity has not been fully characterised in *C. nivalis*, despite the bioprocessing potential associated with the robustness to environmental changes and low temperature requirement of this microalgae. The effects of nutrient deprivation on lipid profile [19] and TAG accumulation [20] have been investigated to reveal significant shifts in lipid composition, and enhanced TAG production, respectively. There are limited studies on lipid production in *C. nivalis* under saline conditions [21–23], with none focussing solely on the neutral lipids or TAGs, which accumulate in lipid bodies. In contrast to *C. reinhardtii*, existing studies of *C. nivalis* provide evidence that salt stress leads to lipid accumulation: Lu et al. [22] used Nile Red as a screening technique in *C. nivalis*, and found that under a range of salt stresses, the highest neutral lipid content (68-fold higher than the control, as measured by fluorescence) was found in 0.17 M NaCl at 7 h, and the highest polar lipid was found in 0.21 M NaCl at 5 h (tenfold higher than the control). Lipidomic analysis has led to identification of a specific subset of lipids as biomarkers of salt stress in *C. nivalis*. These alterations in lipid profile are proposed to reflect the active maintenance of membrane stability by reducing permeability to salt [21, 22]. One study into a related Antarctic *Chlamydomonas* species investigated lipid accumulation under salt stress [24] and found a 23% lipid content (w/w) in 0.27 M NaCl, compared with approximately 15% lipid content at 0.55 M NaCl (sea water). Accumulation also occurred at higher salinities of 1.1–2.2 M NaCl, with 20% lipid content reached under 2.2 M NaCl. Thus, increased salinity influences lipid profile. There are no proteomics studies of *C. nivalis* to date, although there has been a proteomic study (using 2-DE and subsequent MALDI-ToF–MS) on the effect of low-temperature stress (4–6 °C compared with control condition 6–8 °C) on a related Antarctic "*Chlamydomonas* sp." [25].

The physiological responses of *C. reinhardtii* and *C. nivalis* to salt stress are distinct, and to date, there is little mechanistic insight into how they differ. Although quantitative proteomics is not precise proof of physiological function, it does provide a unique profile of metabolic function, and hence provides the first insight into the molecular differences between *C. reinhardtii* and *C. nivalis* when responding to salt stress. To date, no proteomic studies on *C. nivalis* have been published. This study aimed to first quantify any fatty acid accumulation, which was used as a proxy measure of lipid accumulation, in response to salt stress in both *Chlamydomonas* species, and then quantify the associated proteomic shifts to provide insights into the lipid trigger. 8-plex iTRAQ (isobaric tags for relative and absolute quantification) was selected as the method for quantitative proteomic analysis. This method offers the advantage of enabling simultaneous identification and relative quantification of proteins in a multiplex format [26], thus enabling inclusion of two biological replicates for 4 sample conditions. This format was used to study and compare the regulatory proteomic changes of the two species exhibiting different fatty acid accumulation responses under the same environmental stressor. This approach enabled dissection of proteins associated with fatty acid accumulation and those associated purely with stress response.

## Results and discussion

### The impact of salt stress on the physiology of *Chlamydomonas reinhardtii* and *Chlamydomonas nivalis*

A comparison of no additional salt ("0 M NaCl") and addition of 0.2 M NaCl to the media ("0.2 M NaCl") were used to compare control conditions to salt stress. 0.2 M NaCl was selected as a salt level that would not be too toxic and kill the cells, based on our previous preliminary data (see Figures S1 and S2 in Additional file 1) and consistent with other studies [27, 28]. Growth rate, chlorophyll content, carbohydrate content, fatty acid content and photosynthesis rates were used to evaluate the two species' responses to salt.

**Fig. 1:**
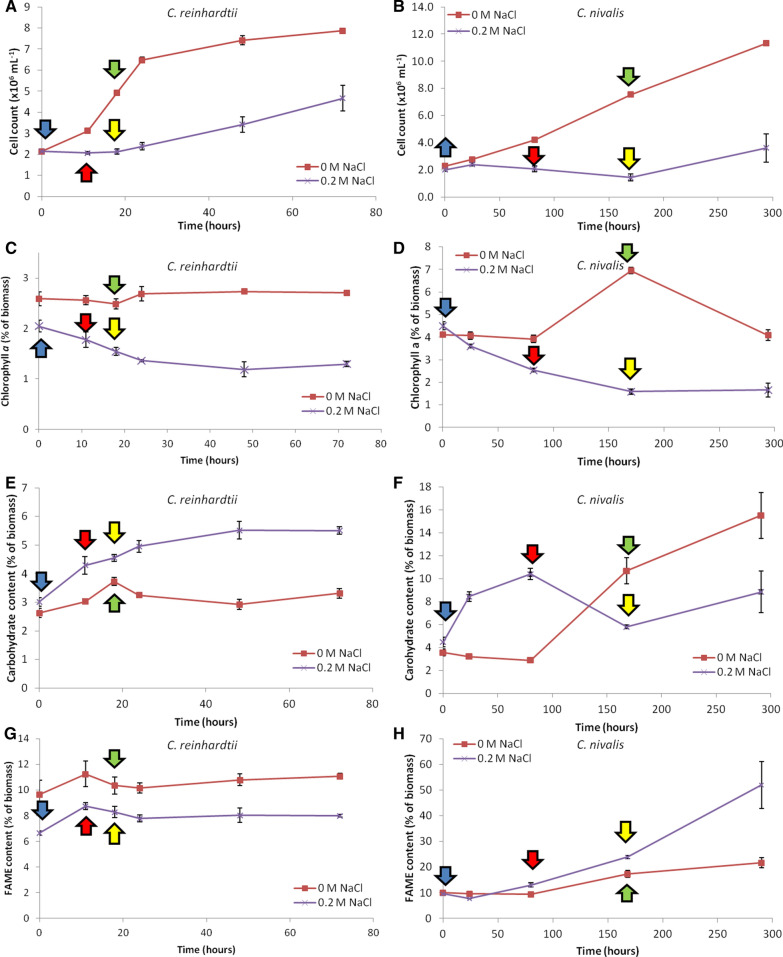
Growth curves, chlorophyll, carbohydrate and FAME content of each strain during salt stress. Growth curves are based on cell count (**A** and **B**), chlorophyll *a* content as a percentage of biomass (**C** and **D**), carbohydrate content as a percentage of biomass (**E** and **F**) and total FAME content as a percentage of biomass (**G** and **H**), for *C. reinhardtii* grown in 0 and 0.2 M NaCl (**A**, **C**, **E**, **G**) and *C. nivalis* grown in 0 and 0.2 M NaCl (**B**, **D**, **F**, **H**). For all experiments n = 3. Error bars show SEM. Arrows indicate the proteomic sampling points (blue arrows: 0 h salt stress; red arrows: early stage salt stress; green arrows: control cultures mid-log phase; yellow arrows: mid-log salt stress). Note that different scales are used for the y axis of the comparative figures

Growth analyses based on cell density showed that the growth rates of both species were detrimentally impacted by the presence of 0.2 M NaCl (Fig. 1A, B). In *C. reinhardtii*, there was still evidence of growth, albeit at a reduced rate (1.8-fold reduction) in salt conditions; the average growth rate was 1.08 × 10^6^ cells day^−1^ under salt stress compared with 1.93 × 10^6^ cells day^−1^ under control conditions. *C. nivalis* showed a more pronounced, 5.6-fold reduction in growth rate; the average growth rate was 1.32 × 10^5^ cells day^−1^, compared with 7.47 × 10^5^ cells day^−1^ under 0 M NaCl. Salt stress can decrease chlorophyll levels in microalgae [29] and this was observed in both species (Fig. 1C, D). The data suggest that both divert resources away from chlorophyll synthesis in response to salt stress.

There was also evidence that *C. reinhardtii* and *C. nivalis* were responding to salt stress by diverting resources to carbohydrates (shown as a percentage of biomass) (Fig. 1E, F). The sustained increase in carbohydrate in *C. reinhardtii* was smaller overall relative to *C. nivalis* (Fig. 1E). In *C. nivalis*, the amount of carbohydrate as a percentage of biomass increased rapidly in cells grown in 0.2 M salt from 4.5 to 10.4% of DCW over 80 h, then decreased closer to initial levels at 168 h (Fig. 1F). This is also the time point where a rapid increase in FAME content was quantified for *C. nivalis* (Fig. 1H). Increasing the amount of carbohydrate stored as starch in response to physiological stress has been seen previously in *C. reinhardtii* cells, for example in nitrogen stress conditions [12]. This is consistent with algal cells increasing starch as a short-term response to stress. This results in storage of carbon assimilated from CO_2_, before being broken down and used in the biosynthesis of neutral lipids [30, 31].

In this experiment, we have not distinguished between starch and other types of carbohydrates, but the observed increase matches those found in other species, which form starch grains as storage molecules. Hence, it is likely that *C. nivalis* is able to respond to salt stress through both the short-term strategy of energy storage in the form of starch, followed by the longer term mechanism of diverting resources to FAME production (indicated by the large increase in FAME content in Fig. 1H). By contrast, salt stress did not cause significant fatty acid accumulation in *C. reinhardtii* (Fig. 1G), which is often the longer term response associated with green algae when shifted to unfavourable environmental conditions such as nitrogen deprivation [12]. *C. nivalis* thus shows a distinct response from *C. reinhardtii*, which is of potential value as a fatty acid producer in salt stress conditions.

**Fig. 2:**
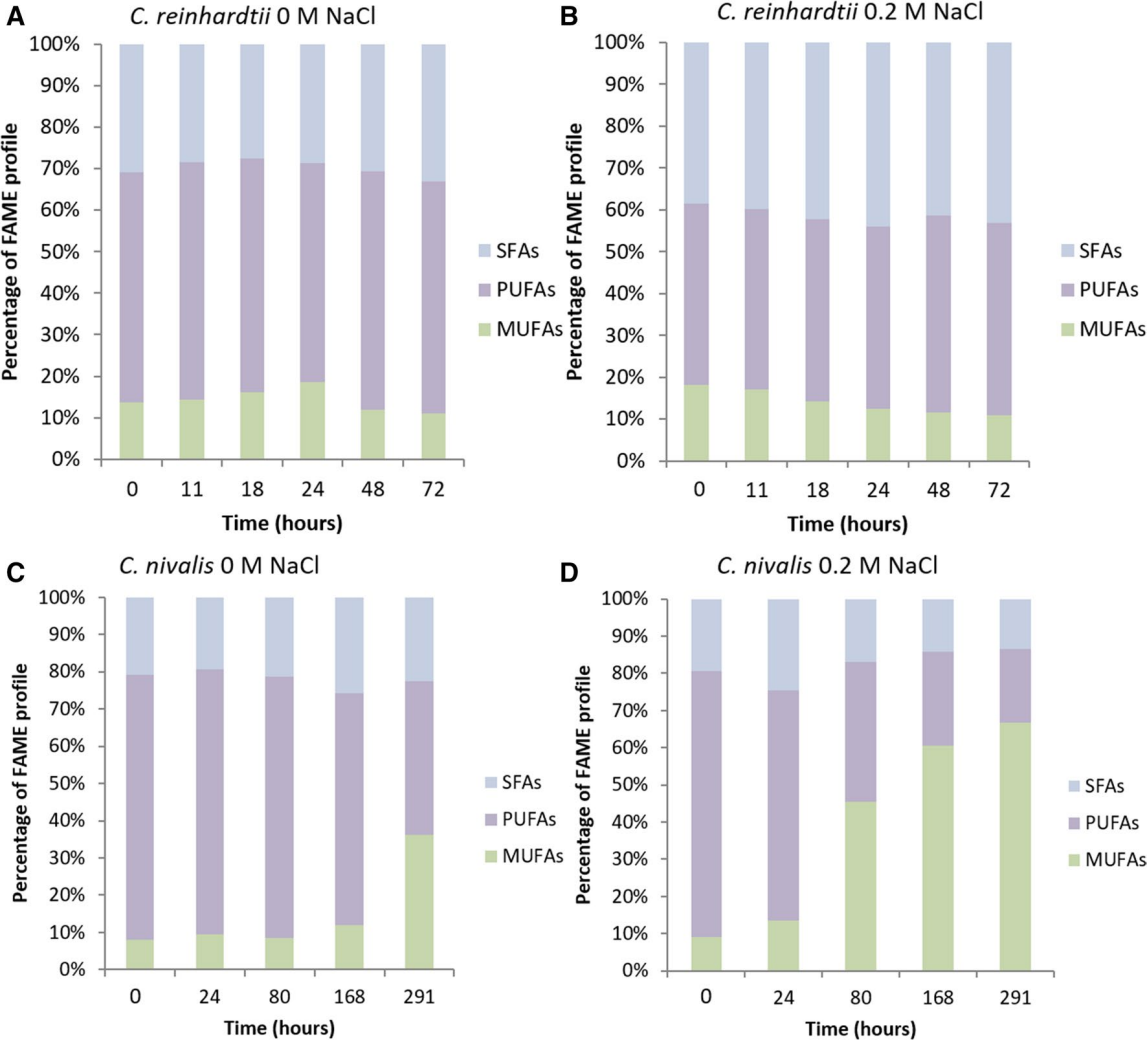
Relative percentages of FAME chain types in each strain during salt stress. FAME chain type relative percentages (in terms of degree of unsaturation) are shown for each time-course experiment for 0 M (**A**) and 0.2 NaCl (**B**) in *C. reinhardtii* and 0 M (**C**) and 0.2 M (**D**) NaCl in *C. nivalis* (n = 3). SFAs = saturated fatty acids; PUFAs = polyunsaturated fatty acids; MUFAs = monounsaturated fatty acids

FAME composition analysis showed that during 0.2 M NaCl stress, there was a slight shift towards a higher percentage of saturated fatty acids in *C. reinhardtii* (Fig. 2A, B, see also Figure S3 in Additional file 1). In *C. nivalis*, FAME analysis showed a significant shift towards monounsaturated fatty acids (MUFAs) (as a percentage of the profile) in 0.2 M NaCl, at the detriment of mostly polyunsaturated fatty acids (PUFAs) (Fig. 2C, D, see also Figure S4 in Additional file 1). It has been shown in a previous study that free radicals, such as ROS, are induced by salt stress and cause degradation of polyunsaturated fatty acids in membranes [32], which are more susceptible to peroxidation than saturated fatty acids (SFAs) [33]. The reduction in PUFAs can therefore be partly explained by the detrimental impacts of salt stress, and partly by increases in lipid storage molecules. *C. nivalis* FAME composition has previously been shown to shift under salt stress with a relative decrease in PUFAs in relation to MUFAs [22], and with increases in C16:0, C16:1, C18:0, C18:1cis [23]. The increase in MUFAs, and in particular C18:1 oleic acid, has also previously been seen in "*Scenedesmus rubescens*-like microalga" under nitrogen deprivation (430% increase) [34], under which the neutral lipids of storage molecules are increased. Similarly, Rao et al. [8] found a large increase in C16:0 and C18:1*cis* under increased salinity conditions in *B. braunii*, with the biggest increase found in C18:1*cis*, which is similar in response to *C. nivalis*. C18:1*cis* is one of the main storage molecules in TAGs [35] and also increases in *N. oculata* under nutrient starvation [36]. Since chloroplasts contain polyunsaturated fatty acids [37], a move away from PUFAs to MUFAs and SFAs is consistent with reduction of these chloroplast lipids, and an increase in storage lipids which occurs when photosynthesis is reduced [35].

As *C. nivalis* demonstrates significant shifts in the lipid profile during lipid accumulation, analysis of the effect that the shifts had on the biofuel properties of the algal oil extracts were carried out and summarised in Table 1, using calculations as detailed in Karpagam et al. [38]. The salt stressed samples demonstrated lower CFPP and LCSF values than control samples, resulting in better fuel performance at cold temperatures, which is advantageous in cold climates. Lower iodine values (IV) and lower degrees of unsaturation are related to lower NO_x_ emissions, therefore the reduction in IV and degree of unsaturation from salt stress is advantageous for biodiesel production. Additionally, higher cetane numbers are better, indicating the ignition qualities of the fuel. Therefore, the salt-stressed cultures have better (higher) cetane number values, especially at the end of the time-course. In this case, the saponification values were similar throughout the time-course and between the conditions, being slightly lower in salt-stressed cultures towards the end of the time-course. Overall, salt stress conditions are shown to improve the quality of biofuel from *C. nivalis*.

**Table 1:** Biofuel properties of *C. nivalis* samples (n = 3)

Sample	Saponification number (mg KOH g^−1^ oil)		Iodine number (g *I*_*2*_ 100 g^−1^ oil)	Cetane number	Degree of unsaturation (wt. %)	Long chain saturation factor (LCSF) (°C)	Cold filter plugging point (CFPP) (°C)
Time (hours)	NaCl concentration (M)						
0	0	193.6 ± 0.1	144.5 ± 0.4	42.0 ± 0.1	117.2 ± 0.4	12.2 ± 0.5	21.9 ± 1.7
0	0.2	193.5 ± 0.0	143.8 ± 0.2	42.1 ± 0.1	115.7 ± 0.2	13.1 ± 0.1	24.6 ± 0.3
24	0	193.8 ± 0.2	145.5 ± 0.5	41.7 ± 0.1	117.2 ± 0.8	12.5 ± 0.4	22.7 ± 1.2
24	0.2	193.8 ± 0.1	131.5 ± 1.2	44.9 ± 0.3	110.6 ± 1.1	11.0 ± 0.2	18.2 ± 0.8
80	0	193.6 ± 0.1	144.2 ± 0.7	42.0 ± 0.1	117.9 ± 0.3	11.6 ± 0.3	20.0 ± 0.8
80	0.2	192.4 ± 0.1	115.9 ± 0.8	48.6 ± 0.2	107.2 ± 0.4	6.8 ± 0.2	4.8 ± 0.7
168	0	194.1 ± 0.1	132.1 ± 0.5	44.7 ± 0.1	113.0 ± 0.3	10.1 ± 0.1	15.2 ± 0.5
168	0.2	191.8 ± 0.1	104.9 ± 0.4	51.2 ± 0.1	104.1 ± 0.1	4.2 ± 0.1	− 3.4 ± 0.4
291	0	193.6 ± 0.3	115.5 ± 1.5	48.5 ± 0.4	107.2 ± 0.7	6.3 ± 0.3	3.4 ± 0.9
291	0.2	191.6 ± 0.1	98.8 ± 0.8	52.6 ± 0.2	101.1 ± 0.7	3.5 ± 0.2	− 5.4 ± 0.8

### Proteomic studies of salt stress in *C. reinhardtii* and *C. nivalis*

**Fig. 3:**
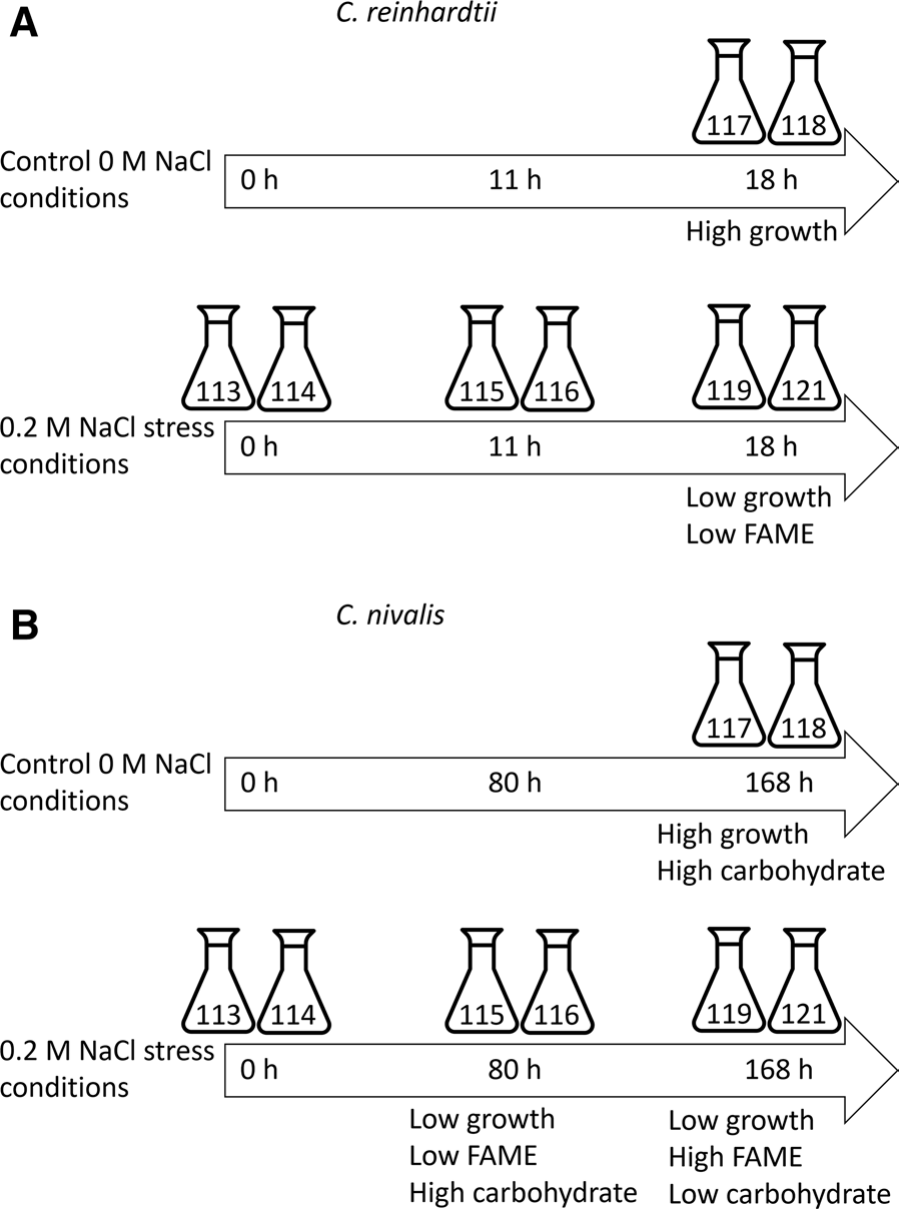
Quantitative proteomic analysis experimental set-up using 8-plex iTRAQ. Two 8-plex iTRAQ experiments were performed for *C. reinhardtii* (**A**) and *C. nivalis* (**B**). Four conditions (time 0 salt stress, early log-phase salt stress, mid-log phase salt stress, and time-matched mid-log control), two biological replicates of each, were compared for each species. The assigned iTRAQ labels are shown for each condition and replicate. Time points were selected at which *C. nivalis* was producing carbohydrate (early log) and fatty acids (mid-log) in response to 0.2 M NaCl stress, with the equivalent points of the growth curve selected for *C. reinhardtii*

A protein profiling approach was taken, to holistically determine alterations in protein changes in response salt stress, rather than focusing on specific pathways; an approach that has yielded valuable insights into lipid accumulation under nitrogen stress [39]. Figure 3 shows the experimental design of the two 8-plex iTRAQ quantitative proteomic experiments for *C. reinhardtii* (Fig. 3A) and *C. nivalis* (Fig. 3B). The main comparisons within each sample group were made to identify proteomic changes associated with the key phenotypic changes of impaired photosynthetic activity, reduced cell division, carbohydrate accumulation and fatty acid accumulation.

#### Proteomic study in *C. reinhardtii* and *C. nivalis*

Biochemical analyses in “The impact of salt stress on the physiology of *Chlamydomonas reinhardtii* and* Chlamydomonas nivali*” Section determined the time point selection of proteomic samples for each species. For the *C. nivalis* proteomic analysis, a time-course experiment of fatty acid producing conditions at 0, 80 and 168 h in 0.2 M NaCl conditions as well as the 168 h time point of the control condition, were compared. The aim was to test the effect of salt stress over time on *C. nivalis,* particularly at the points of rises in carbohydrate and fatty acids, and to compare arrested growth in salt conditions with mid-log growth in control conditions. The corresponding time-points for *C. reinhardtii* were 0, 11 and 18 h.

In terms of protein identification: in *C. reinhardtii*, 139,093 MS and 168,249 MS/MS scans were obtained from the samples to find 27,275 PSMs that corresponded to 3276 identified proteins. A total of 2789 PSMs were found corresponding to 1018 proteins from 279,007 MS and 264,461 MS/MS scans for the *C. nivalis* experiment. 885 proteins were detected and quantified in both iTRAQ experiments (i.e. in both species), using PSM FDR 1% and two or more unique peptides. In addition to these proteins found in both species, 2391 proteins were solely detected in the *C. reinhardtii* experiment and 133 proteins were found in the *C. nivalis* experiment that were not detected in *C. reinhardtii.* The large overlap shows that there is a good basis for comparing the two datasets directly.

Proteins that were statistically different in abundance using ANOVA (p < 0.05) across the treatments are summarised in Tables 2 and 3 for *C. reinhardtii* and *C. nivalis*, respectively. Principal component analysis (PCA) (see Figures S5 and S6, Additional file 1) of the 8-plex iTRAQ-labelled samples show co-clustering of biological replicates for each condition. The greatest difference is between mid-log control samples and salt-stressed samples indicating effects of salt stress at the proteomic level. The 80- and 168-h salt-stressed samples are the most similar, with groupings that overlap.

**Table 2:** Summary of protein changes detected between sample groups in *C. reinhardtii* iTRAQ experiment

Comparison	Number of proteins changed	Number of proteins increased	Number of proteins decreased
0 h and 11 h salt conditions	171	91	80
11 h and 18 h salt conditions	217	94	123
0 h and 18 h salt conditions	212	76	136
18 h control and 18 h salt conditions	337	113	224

**Table 3:** Summary of protein changes detected between sample groups in *C. nivalis* iTRAQ experiment

Comparison	Number of proteins changed	Number of proteins increased	Number of proteins decreased
0 h and 80 h salt conditions	143	115	28
80 h and 168 h salt conditions	22	12	10
0 h and 168 h salt conditions	155	26	129
168 h control and 168 h salt conditions	231	43	188

Patterns of changes and proteins of note are discussed below and are detailed in Table 4, which summarises significant fold changes in notable proteins with a focus on those related to stress tolerance, photosynthesis, carbohydrate metabolism and lipid metabolism in both species. KEGG maps are also provided in Figures S7 and S8 in Additional file 1, to illustrate trends in these protein changes.

**Table 4:** Summary of protein changes in each strain during salt stress

Protein accession ID	Protein name	Pathway/role	Salt stress treatment comparisons with relative protein changes (values indicate fold change)							
			*C. reinhardtii*				*C. nivalis*			
			0–11 h salt	11–18 h salt	0–18 h salt	18 h control–18 h salt	0–80 h salt	80–168 h salt	0–168 h salt	168 h control–168 h salt
*Stress proteins*										
A8HYV3/ Q39603	Heat shock protein 70B	Chloroplast localised heat shock protein protecting against photoinhibition [84]	↑ 1.073	→	↑ 1.077	→	↓ -1.340	→	↓ -1.363	↓ -1.708
A8J3P5	Heat shock protein 70E	Cytosolic heat shock protein [84]	→	→	→	↓ -1.172	→	→	→	→
A8JEU4	Heat shock protein 70A	Cytosolic heat shock protein [84]	→	→	→	→	↓ -1.358	→	↓ -1.334	↓ -1.880
A8IZU0	Heat shock protein 70C	Mitochondrial heat shock protein [84]	→	→	→	→	↓ -1.336	→	↓ -1.386	↓ -1.731
A8J1U1	Heat shock protein 90A	Cytosolic heat shock protein [85]	→	→	→	→	→	→	↓ -1.189	↓ -1.848
Q66T67	Heat shock protein 90C	Chloroplastic heat shock protein [85]	→	→	→	→	→	→	↓ -1.543	↓ -1.868
A8I7T1	Heat shock protein 90B (Fragment)	ER localised heat shock protein [86]	→	→	→	→	→	→	→	↓ -1.771
Q1RS84	Aldehyde-alcohol dehydrogenase	Aldehyde scavenging (during lipid peroxidation) [42]	↑ 1.134	→	↑ 1.106	→	→	→	→	→
A8JHR9/ A8JHS0/ P49644	Glyceraldehyde-3-phosphate dehydrogenase	Glycolysis (and carbohydrate metabolism)	→	↑ 1.155	↑ 1.284	→	↑ 2.166	→	↑ 2.200	→
A8HXA6	Glutathione reductase	Reduction of glutathione disulphide [45]	↓ -1.195	↑ 1.201	→	→	→	↑ 1.277	→	→
A8JG06	Programmed cell death protein 6-interacting protein	Protein self-association and ubiquitin binding	→	→	→	→	→	↑ 1.208	→	→
A8J1C1	Ubiquitin-activating enzyme E1	Ubiquitin conjugation cascade	↓ -1.088	↑ 1.169	→	→	→	↑ 1.254	↑ 1.198	↑ 1.097
A8J5Z8	Aldehyde dehydrogenase	Aldehyde detoxification, ROS scavenging, inhibits lipid peroxidation	→	↑ 1.180	↑ 1.274	↑ 1.271	→	→	↑ 1.306	→
A8JB85	Autophagy-related protein	Autophagy	↑ 1.385	→	↑ 1.274	→	→	→	↑ 1.352	→
A8J431	Stress-related chlorophyll a/b binding protein 2	Non-photochemical quenching, protect photosynthetic apparatus from photo-oxidative damage	→	→	→	↑ 1.776	→	→	↑ 1.572	→
*Photosynthesis proteins*										
Q39588	Carbonic anhydrase, alpha type	Carbon dioxide fixation [87]	→	→	↓ -1.202	↓ -1.135	→	→	→	→
Q8S567	Chlorophyll a/b-binding protein	Photosynthesis and light harvesting	↓ -1.190	→	↓ -1.191	→	→	→	→	→
A8J270	Chlorophyll a-b binding protein of LHCII	Light harvesting in photosystem I	→	→	→	↑ 1.144	→	→	→	→
Q8S3T9	Chlorophyll a-b binding protein of LHCII	Light harvesting in photosystem I	→	→	→	↑ 1.171	→	→	→	→
A8HP84/ P50362	Glyceraldehyde-3-phosphate dehydrogenase	Photosynthesis, Calvin cycle	→	→	→	→	↓ -1.529	→	↓ -1.682	↓ -2.147
Q75VY7	Light-harvesting chlorophyll-a/b protein of photosystem I	Chlorophyll binding, light harvesting in photosystem I	↓ -1.240	→	→	↓ -1.347	→	→	→	→
A8ITV3	Light-harvesting protein of photosystem I	Chlorophyll binding, light harvesting in photosystem I	↓ -1.190	→	↓ -1.191	→	→	→	→	→
Q93VE0	Light-harvesting chlorophyll-a/b binding protein LhcII-4	Chlorophyll binding, light harvesting in photosystem I	→	→	→	↑ 1.147	→	→	→	→
A8J0B1	Low-CO2-inducible protein	Inorganic carbon uptake [52]	↓ -1.161	→	→	→	→	→	→	→
A8J225	Low-CO2-inducible protein (Fragment)	Inorganic carbon uptake [52]	↑ 1.285	→	↑ 1.240	↑ 1.385	→	→	→	→
Q75NZ1	Low-CO2 inducible protein	Inorganic carbon uptake [52]	→	↑ 1.067	→	→	→	→	→	→
A8IT08	Low-CO2-inducible chloroplast envelope protein	L-orthinine transmembrane transport activity	→	→	↑ 1.268	↑ 1.390	→	→	→	→
A8JEV1	Oxygen evolving enhancer protein 3	Photosynthetic electron transfer chain, calcium ion binding	→	↓ -1.150	↓ -1.141	↓ -1.226	↓ -1.495	→	↓ -1.674	→
A8J0E4	Oxygen-evolving enhancer protein 1 of photosystem II	Photosystem II assembly and stability, oxygen evolving activity	→	↓ -1.094	↓ -1.145	↓ -1.290	↓ -1.864	→	↓ -2.186	↓ -2.300
A8IYH9	Oxygen-evolving enhancer protein 2 of photosystem II	Photosynthesis, calcium ion binding	→	→	↓ -1.136	↓ -1.108	↓ -1.520	→	↓ -1.626	↓ -2.072
P12852	Oxygen-evolving enhancer protein 3, chloroplastic	Photosynthesis, calcium ion binding	→	↓ -1.150	↓ -1.141	↓ -1.226	↓ -1.495	→	↓ -1.674	→
Q00914	Photosystem I iron-sulphur centre	Photosynthetic electron transport in photosystem I	→	→	→	→	↓ -1.672	→	↓ -1.932	→
P13352	Photosystem I reaction centre subunit VI, chloroplastic	Photosynthesis	→	→	→	→	→	→	→	↓ -2.285
Q5NKW4	Photosystem I reaction centre subunit II, 20 kDa	Photosynthesis	→	↓ -1.177	→	→	→	→	→	→
Q39615	Photosystem I reaction centre subunit II, chloroplastic	Photosynthesis	→	↓ -1.177	→	→	→	→	→	→
P12154	Photosystem I P700 chlorophyll a apoprotein A1	Photosynthesis, electron transfer, chlorophyll binding	→	→	↓ -1.261	→	↓ -1.823	→	↓ -1.864	→
P09144	Photosystem I P700 chlorophyll a apoprotein A2	Photosynthesis, electron transfer, chlorophyll binding	→	→	↓ -1.200	→	↓ -1.643	→	↓ -1.614	→
P10898	Photosystem II CP43 reaction centre protein	Photosynthetic electron transport in photosystem II, chlorophyll binding	→	→	→	→	↓ -1.623	→	↓ -1.824	↓ -1.469
P37255	Photosystem II CP47 reaction centre protein	Photosynthetic electron transport in photosystem II, chlorophyll binding	→	→	→	→	↓ -1.583	→	↓ -1.693	→
P06007	Photosystem II D2 protein	Photosynthetic electron transport in photosystem II, chlorophyll binding	→	→	→	→	↓ -1.611	→	↓ -1.634	→
P07753	Photosystem II protein D1	Photosynthetic electron transport in photosystem II, chlorophyll binding	→	→	→	→	↓ -1.840	→	↓ -1.727	↓ -1.445
A8J4S1	Photosystem I reaction centre subunit III	Photosynthesis, protein domain specific binding	→	↓ -1.255	↓ -1.289	↓ -1.461	→	→	→	→
P12356	Photosystem I reaction centre subunit III, chloroplastic	Photosynthesis, protein domain specific binding	→	↓ -1.255	↓ -1.289	↓ -1.461	→	→	→	→
A8HVJ9	Photosystem II stability/assembly factor HCF136 fragment	Photosystem II biogenesis	→	→	→	↓ -1.190	→	→	→	→
A8JGP9	Ribulose bisphosphate carboxylase small chain	Carbon fixation, photorespiration, photosynthesis	↓ -1.213	→	↓ -1.356	→	→	→	→	→
P00873	Ribulose bisphosphate carboxylase small chain 1, chloroplastic	Carbon fixation, photorespiration, photosynthesis	↓ -1.213	→	↓ -1.356	→	→	→	→	→
P08475	Ribulose bisphosphate carboxylase small chain 2, chloroplastic	Carbon fixation, photorespiration, photosynthesis	↓ -1.219	→	↓ -1.357	→	→	→	→	→
P00877	Ribulose bisphosphate carboxylase large chain	Photorespiration, reductive pentose-phosphate cycle	→	↓ -1.078	↓ -1.154	↓ -1.110	↓ -1.810	→	↓ -1.991	↓ -2.751
P23489	Ribulose bisphosphate carboxylase/oxygenase activase, chloroplastic	Photosynthesis regulation	→	↓ -1.107	↓ -1.108	↓ -1.186	→	→	↓ -1.555	↓ -2.809
Q6SA05	Rubisco activase	Photosynthesis regulation	→	↓ -1.107	↓ -1.108	→	→	→	↓ -1.555	↓ -2.809
*Carbohydrate proteins*										
Q2VA40	Alpha-1,4 glucan phosphorylase	Starch degradation [88]	↑ 1.123	↑ 1.105	↑ 1.240	↑ 1.122	↑ 1.237	↑ 1.158	↑ 1.433	↓ -1.688
Q2VA41/ A8IYK1	Alpha-1,4 glucan phosphorylase	Starch degradation [88]	↑ 1.096	→	↑ 1.147	→	→	→	→	→
A2PZC2	UDP-glucose:protein transglucosylase	Polysaccharide biosynthesis	→	→	→	→	↓ -1.535	→	↓ -1.713	↓ -2.510
A8HW52	Starch branching enzyme	Starch biosynthesis	→	→	→	→	→	→	→	↓ -2.318
A8IHX1	Starch branching enzyme	Starch biosynthesis	→	→	↑ 1.207	→	→	→	→	→
A8J914	UDP-glucose 6-dehydrogenase	Starch catabolism for glycolysis [58]	↑ 1.263	↑ 1.239	↑ 1.562	↑ 1.478	→	→	→	→
A2PZC3	UDP-glucose 6-dehydrogenase	Starch catabolism for glycolysis [58]	↑ 1.298	↑ 1.231	↑ 1.595	↑ 1.461	→	→	→	↑ 2.008
*Lipid proteins*										
A8HNN4/ A0A0B5KTL4	Glycerol-3-phosphate dehydrogenase	Glycerol metabolism, link between carbohydrate and lipid metabolism	↑ 1.675	↑ 1.169	↑ 1.959	↑ 1.959	↑ 2.135	↑ 1.192	↑ 2.544	↑ 2.648
A0A0B5KYA7	Glycerol-3-phosphate dehydrogenase	Glycerol metabolism, link between carbohydrate and lipid metabolism	↑ 1.719	↑ 1.179	↑ 2.027	↑ 2.064	→	→	→	→
A8HNN6	Glycerol-3-phosphate dehydrogenase (fragment)	Glycerol metabolism, link between carbohydrate and lipid metabolism	↑ 1.744	↑ 1.189	↑ 2.073	↑ 2.109	→	→	→	→
A8J6J6	Acetyl-CoA acyltransferase	Catabolic beta-oxidation of fatty acids [63]	↑ 1.162	→	↑ 1.181	↑ 1.209	→	→	↑ 1.250	↑ 1.375
A8J2S0	Citrate synthase	Tricarboxylic acid cycle	→	→	→	→	→	→	→	↓ -2.725
Q6DN05	Betaine lipid synthase	Betaine lipid synthesis, including DGTS synthesis	→	↓ -1.093	→	→	→	→	→	→
A8HXT4	Pyruvate carboxylase	Pyruvate metabolism, gluconeogenesis, links carbohydrate and lipid metabolism	→	↓ -1.047	↓ -1.100	→	→	→	→	→
A8JGF4	Biotin carboxylase, acetyl-CoA carboxylase component	Malonyl-CoA biosynthesis, lipid metabolism	→	→	→	↓ -1.113	↓ -1.479	→	↓ -1.482	↓ -1.860
Q763T6	UDP-sulfoquinovose synthase	Glycerolipid metabolism, sulfoquinovosyl diacylglycerol (SQDG) metabolism	→	→	→	→	↓ -1.275	→	↓ -1.289	↓ -1.770
A8J1T4	Dihydrolipoyl dehydrogenase	Acetyl CoA production, cell redox homeostasis	→	→	→	→	↑ 1.110	→	→	↑ 1.149

Changes in stress-related proteins, photosynthesis-related proteins, carbohydrate-related proteins and lipid-related proteins are shown for *C. reinhardtii* and *C. nivalis* subjected to salt stress. Arrows indicate up-regulation (↑), down-regulation (↓) and not significantly changed ( →). Values indicate fold changes

#### Salt stress responses

Changes in notable stress-related proteins can help to reveal the extent to which each species experiences salt stress, the mechanisms employed to respond to or tolerate salt stress, and how these responses interact with fatty acid accumulation in each species.

Both species clearly experience stress in response to salt conditions: this is indicated by increases in abundances of programmed cell death protein 6-interacting protein in *C. nivalis*, and by increases in stress-related chlorophyll a/b-binding protein 2, autophagy-related protein, and ubiquitin-acting enzyme in both species. Ubiquitin-acting enzyme has been linked to oxidative stress and the accumulation of ROS in *C. reinhardtii* [40]. Aldehyde dehydrogenase was at higher abundance at mid-log phase salt conditions in *C. reinhardtii*, mirroring the response of *C. nivalis* which showed a higher abundance of this protein at mid-log phase stress. This enzyme detoxifies aldehydes, scavenges ROS and inhibits lipid peroxidation by free radicals, and has been linked to stress tolerance in Arabidopsis [41]. The greater increase of this enzyme in *C. reinhardtii* over time (showing increased abundance in 3 of 4 comparisons), compared to *C. nivalis* implies that ROS may be a greater problem in *C. reinhardtii* in response to salt. This is supported by a higher abundance of bi-functional enzyme aldehyde-alcohol dehydrogenase (Q1RS84) between inoculation and early/mid-log phase salt stress in *C. reinhardtii.* This enzyme has been linked to mitigation of oxidative stress as it acts as an aldehyde scavenger during lipid peroxidation [42]. It is shown to coincide with starch accumulation, reinforcing that the starch increase in *C. reinhardtii* is part of a stress response, although it can be synthesised in starchless mutants at levels similar to the wild type [43]. The abundance was unchanged in *C. nivalis,* suggesting it is not required due to a potentially lower incidence of ROS and lipid peroxidation. By contrast, an enzyme involved with glycolysis and the production of energy for cellular metabolism, glyceraldehyde-3-phosphate dehydrogenase (cytosolic form), had a higher abundance at mid-log phase in *C. reinhardtii*, and at early and mid-log phase in *C. nivalis*. It has also been suggested that this enzyme plays a role in Arabidopsis in the mediation of ROS signalling during stresses such as drought or salinity stress, and can aid the plants in adaption to salinity or other types of stress [44].

Both species need to employ oxidative stress mitigation mechanisms in response to salt stress, even though *C. nivalis* is more salt stress tolerant than *C. reinhardtii*. Glutathione reductase, a protein that acts as an antioxidant removing toxic free-radicals in *C. reinhardtii* [45], was at a higher abundance in *C. nivalis* cultures at the onset of fatty acid accumulation in early to mid-log phase. There was a similar temporal salt response in *C. reinhardtii,* which also showed a higher abundance of this protein at mid-log phase salt stress. This followed an initial decrease from inoculation to early log phase salt. The data suggest that *C. reinhardtii* might be suffering greater initial damage due to down-regulation of this stress response mechanism. *C. nivalis* may therefore be more able than *C. reinhardtii* to direct resources towards important oxidative stress mitigation over other processes. For example, *C. reinhardtii* shows early increased abundance in many lipid and carbohydrate processes where *C. nivalis* does not.

Lastly, when addressing stress-related proteins, heat shock proteins may reveal not only the extent to which each species experiences stress, but also mechanisms that each species has for responding to salinity stress. *C. reinhardtii* showed a higher abundance of heat shock protein (HSP) 70B at mid-log phase than early log phase salt stress, although a lower abundance of heat shock protein 70E (A8J3P5) when comparing control and salt-stressed cultures. By contrast, *C. nivalis* showed a lower abundance of several heat shock proteins over the time-course. HSP70B has been shown to increase under irradiance stress conditions in *C. reinhardtii*, whilst other heat shock proteins were down-regulated [46]; HSP70B is of interest due to its role in PSII repair. Later it is discussed that *C. nivalis* downregulates PSII under salt stress but not in *C. reinhardtii*. This may explain why it is unchanged where it is needed in *C. reinhardtii* but decreased in *C. nivalis* where maintenance of PSII is not required. Additionally, HSP70B has previously been shown to increase under salinity stress in salt-adapted *C. reinhardtii* cells, suggesting it may have a role in salinity tolerance [47]. Therefore, whilst the reduced abundance of heat shock proteins in *C. nivalis* may seem unexpected, this phenomenon has been shown to occur in *Chlamydomonas* during stress. It has been proposed to be a by-product of the cell not being able to maintain the biosynthesis of these proteins under salt stress conditions [46]. One explanation for this may be that *C. nivalis* experiences fewer mis-folded proteins due to adaptations to salt, and therefore requires fewer HSPs. This has been explored in other organisms, where HSPs decrease in response to stress. As a consequence, it is theorised that stress adaptation can be achieved through other mechanisms that were less energetically "costly" to the organism than the production of adenosine triphosphate (ATP) binding HSPs [48]. This would be consistent with conservation of ATP, a key metabolite and ‘energy currency’ of cells.

This may therefore provide evidence that *C. nivalis* has better salt-tolerance mechanisms, demonstrated by reduction in need for HSPs. These mechanisms could include altering the lipid profile of cell membranes, as discussed by Lu et al. [21]. The increase of an autophagy-related protein (A8JB85) in both *C. reinhardtii* in the initial stages of salt exposure and *C. nivalis* in the later stages, suggests programmed cell death due to salt stress. In addition, the data are consistent with degradation and recycling of cell components to improve the health of the culture, in an attempt to promote cell survival under stress [49]. It has been shown that inhibition of fatty acid synthesis activates autophagy in *C. reinhardtii* [50]. The lack of accumulation of fatty acid in *C. reinhardtii* in this study suggests that the onset of autophagy is much earlier than in *C. nivalis* where fatty acids accumulate over time. Overall the fact that the indicators of stress in *C. reinhardtii*, such as HSPs and oxidative stress responses, do not occur in *C. nivalis* (or occur at a later time-point) suggests that *C. nivalis* does not appear to be undergoing stress to the same degree as *C. reinhardtii*.

#### Changes in photosynthetic pathways in response to salt stress

Many small subunit RuBisCO proteins were reduced in abundance in *C. reinhardtii* that remained unchanged in *C. nivalis,* contrasting with the response of RuBisCO large subunit (P00877), which has a greater decrease in abundance during salt stress in *C. nivalis* than in *C. reinhardtii* (Table 4). RuBisCO activase also decreased in abundance in both species in response to salt, with the greater decrease in abundance found in *C. nivalis*. Decreases in RuBisCO activity suggest that photosynthetic activity is reduced and deprioritised. RuBisCO activase is required for efficient photosynthesis and reduction of this enzyme indicates that the cells have shifted into a quiescent state, with carbon fixation de-prioritised in favour of other cellular processes. As *C. nivalis* shows greater reduction of RuBisCO, this indicates that early on the cells switch to the quiescent state and start directing their resources into storage molecules. It has previously been found that several photosynthesis-related proteins are down-regulated in model plant species in response to salt stress [51]. This is supported by the low CO_2_-inducible proteins, which increased in abundance in *C. reinhardtii*, whilst remaining unchanged in *C. nivalis.* As these proteins are responsible for inorganic carbon uptake [52], the data suggest *C. reinhardtii* is actively maintaining photosynthetic processes under sub-optimal conditions. In contrast, *C. nivalis* does not need to increase carbon uptake as photosynthesis proteins generally appear decreased. Carbonic anhydrase was decreased in abundance in *C. reinhardtii* but not in *C. nivalis*, showing that salt has a strong negative effect on carbon fixation and carbon concentrating mechanisms that can counter the effects of low organic carbon [52] in *C. reinhardtii*. However, there was also evidence of a reduction in Calvin cycle activity in *C. nivalis, as* a form of glyceraldehyde-3-phosphate dehydrogenase, GAPA (chloroplastic) (P50362) decreases in abundance in *C. nivalis*, but not *C. reinhardtii*. The large decrease in *C. nivalis* RuBisCO proteins suggests a quiescent state. In contrast, *C. reinhardtii* appears to actively maintain carbon fixation, but still experiences low carbon concentrations, likely due to the greater damage done to photosynthetic systems.

Whilst both species show decreases in photosynthetic proteins, the balance of photosystems I and II have been revealed as a key difference between the two species. Photosystem II reaction centre proteins were unaffected in *C. reinhardtii* (with the exception of photosystem II stability/assembly factor HCF136 fragment (A8HVJ9)), but decreased in abundance in *C. nivalis*. Additionally, oxygen evolver enhancer proteins of photosystem II were decreased in both species, with the greater decrease occurring in *C. nivalis*. Photosystem II is more likely to be inactivated than PSI during sub-optimal conditions [53], resulting in photoinhibition [54], suggesting that *C. nivalis* undergoes photoinhibition whilst *C. reinhardtii* does not. In contrast, photosystem I reaction centres were largely decreased in *C. reinhardtii*, whilst remaining unchanged in *C. nivalis*. Likewise, light harvesting proteins of photosystem I decreased in abundance in *C. reinhardtii* whilst remaining unchanged in *C. nivalis*. PSI is less likely than PSII to become damaged, but has a slower recovery than PSII once damage occurs [54]. Active controlled photoinhibition of PSII can regulate the electron transfer chain and protect against ROS formation and therefore against PSI damage [55]. By decreasing PSII, *C. nivalis* protects against this damage whilst *C. reinhardtii* does not. Subsequently, *C. nivalis* maintains PSI proteins whilst *C. reinhardtii* shows a reduction in them, likely due to longer term damage to the photosynthetic apparatus in *C. reinhardtii*. Chlorophyll a-b binding proteins of LHCII increased in abundance in *C. reinhardtii*, whilst remaining unchanged in *C. nivalis*. By contrast, chlorophyll a-b binding proteins of LHCI decreased in *C. reinhardtii*, whilst also remaining unchanged in *C. nivalis*. LHCI only transfers light energy to PSI, but LHCII can transfer light to either PSI or PSII [56], in a phosphorylation-dependent reaction.

#### Changes in carbohydrate-related pathways in response to salt stress

There were few proteins identified in both species that were linked with carbohydrate pathways, but some key proteins involved in starch catabolism and starch synthesis revealed differences in the response (also demonstrated in Figure S8 in Additional file 1). The assay used in this study measured total carbohydrates as an estimation of starch content.

UDP-glucose:protein transglucosylase was lower in abundance over time with salt stress in *C. nivalis*, but unaltered in *C. reinhardtii*. The data suggest polysaccharide synthesis was decreased and resources directed away from starch resources in *C. nivalis*. In addition, starch branching enzyme was lower in abundance at 168 h of salt exposure (decreasing carbohydrate levels) compared with control conditions (increasing carbohydrate levels) in *C. nivalis*. By contrast, *C. reinhardtii* had a higher abundance of this protein at 18 h salt exposure relative to control, which matches the phenotype of a carbohydrate increase.

Starch catabolism is also important, especially as resources are directed from starch storage to lipid storage. Alpha-1,4 glucan phosphorylase, a protein causing the degradation of starch and important in starch remobilisation [57], was higher in abundance in both species in response to salt stress. The highest fold change of 1.43 was seen in *C. nivalis* in the 0–168 h comparison, which matches the decrease in carbohydrate found after 80 h as it is broken down to be used as a resource for fatty acid accumulation. *C. reinhardtii* showed lower fold change increases, suggesting less mobilisation of carbohydrate resources into other processes (including fatty acid accumulation), especially since the initial carbohydrate accumulation in *C. reinhardtii* is lower than *C. nivalis*. Since the *C. reinhardtii* strain used was a low-starch mutant, the initially reduced carbohydrate accumulation would be expected to lead to lower starch catabolism activity. UDP-glucose 6-dehydrogenase, an enzyme that catalyses starch into glucose for use in glycolysis [58], was higher in abundance in 168 h salt conditions compared with a control in *C. nivalis*, which matches the phenotypes observed since carbohydrate was being decreased in salt conditions but rising in control conditions at the compared time-points. Salt exposure caused higher abundance of this protein over time in all *C. reinhardtii* comparisons, although the fold changes were lower than that found in *C. nivalis*.

Overall *C. nivalis* showed larger fold changes than *C. reinhardtii* in proteins involved with starch catabolism, consistent with *C. reinhardtii* being a starchless mutant. The data suggest that salinity stress results in *C. nivalis* diverting starch into fatty acid accumulation.

#### Changes in lipid-related pathways in response to salt stress

Differences found in lipid proteins are particularly interesting, since the key phenotypic difference between the two species is their lipid response, represented by the fatty acid yield changes. In both species and at all time-points, glycerol-3-phosphate dehydrogenase (A8HNN4) was increased in abundance. This enzyme is responsible for the glycerol metabolism, shown to be increased in high salinities [59], and results in the production of compatible solutes. It is also a major linking protein between the carbohydrate and lipid metabolism [60], and increasing this enzyme in plants can result in a significant increase in oil content [61]. Since this enzyme increased in both species, it is clearly important during salt stress, with the primary function as osmotic stress protection, but is not responsible for the differences found in fatty acid accumulation. Pyruvate carboxylase, another enzyme that links carbohydrate and lipid metabolism, decreased in abundance in *C. reinhardtii*, but was unaffected in *C. nivalis*. This may be part of a bottleneck that prevents lipid accumulation taking place in *C. reinhardtii*, and may explain the lack of effect of salt on fatty acid content.

Biotin carboxylase, an essential acetyl-CoA carboxylase (ACC) component, reduced in abundance in both species, but earlier and with a greater fold change in *C. nivalis*. Since acetyl CoA-carboxylase is the committing, rate-limiting step in fatty acid synthesis, which produces malonyl CoA as the fatty acid building block [62], it may be expected that reduction in biotin carboxylase would result in a fatty acid decrease, however it is not shown to be rate limiting here for *C. nivalis*. This is supported by Longworth et al. [12], who demonstrated that down-regulation of biotin carboxylase during nitrogen depletion is associated with lipid accumulation.

Lipid catabolism also plays a key role in accumulation differences: in *C. reinhardtii*, acetyl-CoA acyltransferase (A8J6J6) increased much earlier than *C. nivalis* in response to salt exposure. This enzyme may be involved in catabolic beta-oxidation of fatty acids [63], suggesting there is an increase in the breakdown of fatty acids for energy release for use in the cell from the early stages of salt stress in *C. reinhardtii*, thus preventing fatty acid accumulation. For *C. nivalis*, fatty acid degradation only happens later in the growth cycle, perhaps once resources become more limited due to a decrease in photosynthesis.

One of the most notable protein changes that impacts lipid synthesis is a decrease in citrate synthase in *C. nivalis* at the point of fatty acid accumulation. The decrease in the activity of this enzyme indicates that acetyl CoA is being directed away from the TCA cycle and therefore is available for fatty acid biosynthesis [64]. Overexpression of this enzyme has been shown to drastically reduce TAG content of algae through this redirection of acetyl CoA, and conversely knocking the gene out results in huge TAG increases since the two processes directly compete [65, 66]. This enzyme is therefore a key difference in the direction of resources in relation to fatty acid metabolism. In addition, there is evidence that *C. nivalis* has a higher availability of acetyl CoA under salt stress, since it shows increased abundance of dihydrolipoyl dehydrogenase, which is involved in the production of acetyl CoA, and this protein was detected but unaffected in abundance in *C. reinhardtii*. This therefore suggests a greater availability of the substrate for fatty acid synthesis in *C. nivalis.* The increase in dihydrolipoyl dehydrogenase has been shown to increase TAG accumulation in algae [67]. Thus, the data suggest that *C. nivalis* redirects acetyl CoA to fatty acid synthesis rather than the TCA cycle and energy. *C. nivalis* accumulates TAG as a result.

Alterations in membrane lipid pathways could play a key role in the differences between the two species’ fatty acid responses. For example, betaine lipid synthase was lower in abundance in the later stage of salt stress in *C. reinhardtii*, but remained unaffected in *C. nivalis*. This enzyme is important in the synthesis of DGTS (mainly composed of C18:3 FAs) for lipid membranes of *C. reinhardtii* [68]. It has not previously been found to decrease in this species under salt stress, or under nitrogen deprivation [69], unlike other lipid types. It has been shown in previous research that DGTS decreases in response to salt stress in *C. nivalis*, increasing the degree of saturation of fatty acids and permeability of the membrane [22]. The FAME data show that both species generally have lower C18:3 levels during salt stress than in control conditions, although the differences are small. The proteomic data, however, suggests that *C. reinhardtii* is making the active change in membrane permeability whilst *C. nivalis* is not (Fig. 2). Additionally, UDP-sulfoquinovose synthase (Q763T6) decreased in abundance in salt-stressed *C. nivalis* samples only. This enzyme is involved in glycerolipid metabolism, and also sulfoquinovosyl diacylglycerol (SQDG) metabolism: a major constituent of thylakoid membranes in microalgae. By contrast, NaCl has been shown to trigger the increase of SQDG in a previous *C. nivalis* study, and is thought to stabilise membranes and protein complexes in photosystem II [21]. Findings of photosynthetic protein changes discussed previously showed that PSII proteins were decreased in abundance in *C. nivalis*, and this may allow resources to be directed into lipids instead. SQDG has also been shown to reduce when TAG accumulates under nitrogen deprivation, and it has been theorised that FAs are liberated from SQDG for use in TAG synthesis [65]. Therefore, the finding in this study that this protein was decreased in abundance implies that TAG was favoured over SQDG, which supports the FAME data of fatty acid accumulation in this time-course experiment, and provides potential mechanisms for a key difference between the two species’ fatty acid responses.

When discussing the differences in fatty acid responses of these two species, it should be noted that the differences in the culturing conditions for each species may be considered. Controls have been used for each species in this study to provide a baseline from which to measure relative changes and allow the two species to be compared. Furthermore, the *C. reinhardtii* strain was a low-starch mutant, which will impact the comparison between the two species since one contains a mutation. In each of these cases, the conditions and strains were selected in order to provide the best possible yields for each species. *C. reinhardtii* is well studied, with low-starch mutants available that historically show good lipid accumulation, and therefore would show significant fatty acid accumulation if salt acted as a lipid trigger in this species. *C. reinhardtii* strain CC-4325 sta1-1 mt- [Ball I7] therefore provides a good comparison for the *C. nivalis* strain in terms of fatty acid production capacity. Additionally, mixotrophic conditions would be expected to yield higher fatty acid accumulation than photoautotrophic conditions [70], but this is not what has been observed in this study.

### Photosynthesis and respiration activity

**Fig. 4:**
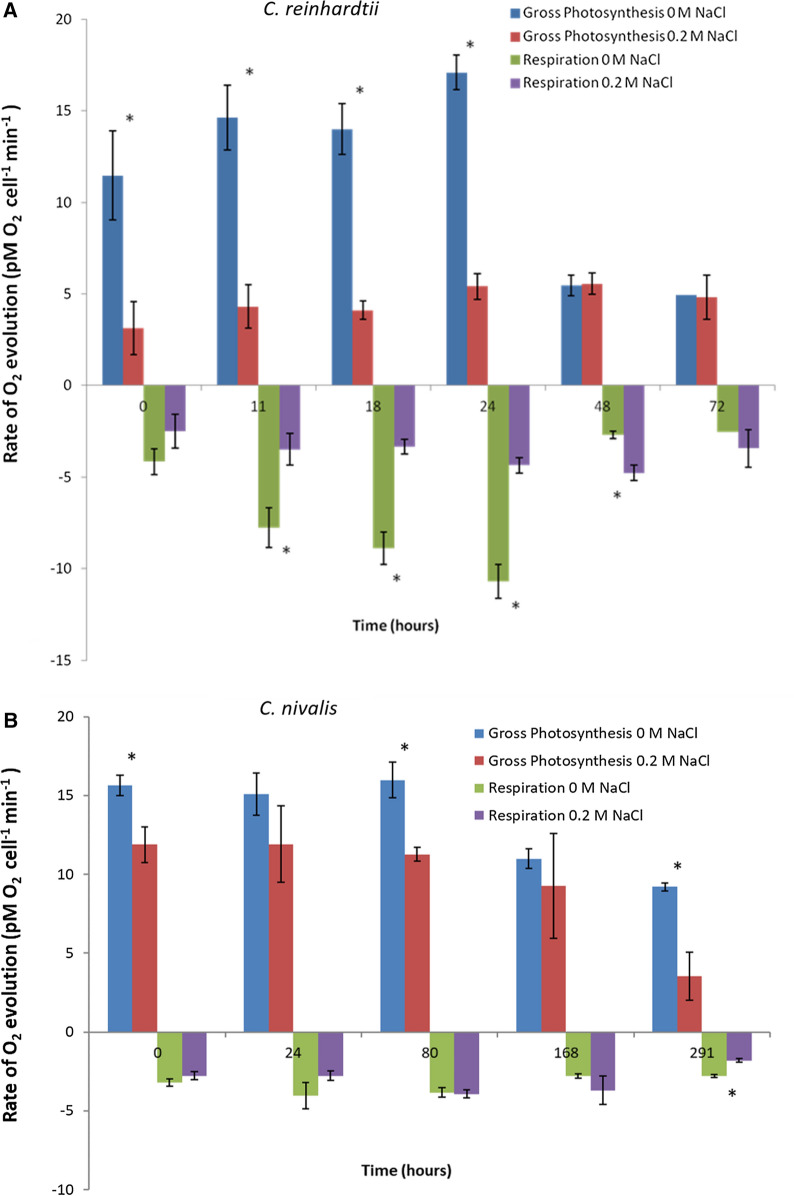
Photosynthetic activity and respiration rates in each strain during salt stress**.** Photosynthesis and respiration rates were measured by rate of oxygen evolution and uptake in *C. reinhardtii* (**A**) and *C. nivalis* (**B**) grown in 0 and 0.2 M NaCl. *T*-tests were carried out to identify statistically significant changes between treatments, "*" indicates a significant result (*p* < 0.05) in the comparison of 0 and 0.2. Error bars show SEM (*n* = 3)

Photosynthetic and respiration rates were calculated as the rate of O_2_ evolution during light and dark conditions, respectively. These were used to further evaluate alterations in response to salinity stress (Fig. 4) and to provide additional data for the photosynthetic protein changes observed in Table 4 and discussed in Sect. “Changes in photosynthetic pathways in response to salt stress”. After 11 h, salinity stress reduced the oxygen evolution rate by 3.4-fold from 14.63 pM O_2_ cell^−1^ min^−1^ in control cultures to 4.31 pM O_2_ cell^−1^ min^−1^ in 0.2 M NaCl (p = 0.0011) in *C. reinhardtii* (Fig. 4A). This occurred during the early exponential growth phase. In comparison, significant changes were measured in *C. nivalis* at the equivalent growth phase (80 h) (Fig. 4B). At this time, control cultures had an oxygen evolution rate of 15.97 pM O_2_ cell^−1^ min^−1^ which was 1.4-fold higher than 11.27 pM O_2_ cell^−1^ min^−1^ in 0.2 M NaCl (*p* = 0.0026), as shown in Fig. 4B. While both species show reduction in photosynthesis rates during salt stress, this is smaller in *C. nivalis* relative to *C. reinhardtii.*

Generally, there appears to be a more abrupt and marked decrease in photosynthesis-related proteins in *C. nivalis* relative to *C. reinhardtii* (Fig. 4), which could allow the cells to focus cellular processes on fatty acid production and go into a stasis-like state. Despite the reduction in RuBisCO, *C. nivalis* retains good photosynthesis rates. *C. reinhardtii* however*,* shows a lower decrease in activity but also fewer mechanisms to cope to with salt stress, in terms of photosystem protection strategies and carbon concentrating mechanisms. The contrast in photosystem I and II balance is an important distinction in the two species’ responses, with *C. reinhardtii* decreasing PSI proteins whilst *C. nivalis* decreases PSII proteins. As PSI proteins take longer to recover once oxidative damage occurs, this would suggest a much larger reduction in photosynthetic activity of *C. reinhardtii*, which is supported by the data shown in Fig. 4. These data are consistent with the quantitative proteomic data and thus provide validation of the proteomic dataset.

### Interaction of salt stress, fatty acid accumulation and algal cell biology

Salt causes two types of stress: ionic stress and osmotic stress [71]. Green microalgae tolerate osmotic stress using osmoregulation and the formation of a compatible solute, which is often glycerol. The source of carbon for the glycerol can come from starch breakdown, since the high salinity inhibits photosynthesis [72]. *C. reinhardtii* may then be continuing to use the available energy and resources to maintain cell functions under increased intracellular ionic or osmotic stress. The up-regulation of many other processes may partly be why fatty acids do not accumulate in this species under salt stress, because other processes are favoured.

Lipid metabolism and carbohydrate metabolism are affected by salt stress (as shown in Figures S7 and S8 in Additional file 1), and many of the phenotypic changes observed can be explained through these regulatory changes. The reduction of fatty acid biosynthesis in *C. reinhardtii* might be attributed to not having effective salt tolerance mechanisms. Due to the fact that *C. reinhardtii* has a greater detrimental response to salt, shown through the large decrease in photosynthetic activity, it is likely that the cell is directing a greater amount of resources into survival, and that fatty acid accumulation is being shut down as an unnecessary part of metabolism.

The maintenance of photosynthetic capacity is important, as this study searched for an alternative lipid trigger to nitrogen deprivation—a trigger that caused photosynthetic capacity to be lost [73]. Part of salt tolerance is the ability to maintain photosynthetic apparatus and repair damage to photosystems, and therefore salt tolerance plays an important role in an effective salt-triggered fatty acid producer.

Differences in the lipid metabolism of the two species in response to salt provide important considerations for the culturing of algae under lipid triggering conditions. The model species, *C. reinhardtii,* showed an early response of increased abundance of lipid degradation proteins under salt conditions, showing that even though a certain condition may trigger lipid accumulation in one species, the toxic effects of salt can cause resources to be used before they can be accumulated in fatty acids. Alternatively, resources may be directed into glycerol production as a compatible solute in *C. reinhardtii*, but not converted to TAGs.

The reason for the "lipid trigger" phenomenon occurring, first documented by Sheehan et al. [74], was suggested to be due to nitrogen deprivation decreasing some key processes including cell division but not affecting lipid production, so lipids accumulate. However, more recently it has been suggested that oxidative stress is linked to lipid production in microalgae [75], and applying oxidative stress leads to increases in lipid content. Since salt can induce oxidative stress, it follows that salt may cause a lipid increase. The occurrence of fatty acid accumulation under salt stress observed in this study may support the theory that it is oxidative stress that causes lipid accumulation, rather than an excess of ATP:AMP ratio as theorised by Botham and Ratledge [76].

## Conclusions

This study is the first to have investigated the physiological changes in *C. nivalis* in response to salt stress with a quantitative proteomics analysis. This algal species has a low optimum temperature for growth, and has therefore generated interest for its use as a biotechnology host strain for lipid production. Although it is an un-sequenced organism, the ability to increase fatty acid content under salt stress, and its relatedness to the model alga *C. reinhardtii* enabled detailed metabolic pathway analysis. In addition to increased overall fatty acid content, profile shifts away from PUFAs and towards increased MUFAs, especially the storage molecule C18:1*cis*, indicating a lipid response to salt stress in *C. nivalis* similar to that found under nutrient stress in other algal strains. By contrast, *C. reinhardtii* did not exhibit the same lipid accumulation response to increased salinity, despite growth, photosynthesis and proteomic data indicating a stress response. Moreover, the analysis of fatty acids for biofuel quality purposes revealed that salt stress improves biofuel quality in *C. nivalis*. The comparative study with *C. reinhardtii* has shown several key physiological and proteome level differences, especially in the relative abundances of PSII and SQDG proteins, which help to elucidate what causes salt stress to be a lipid trigger for one species but not for the other. Whilst both strains experience salinity stress, *C. nivalis* exhibits better tolerance mechanisms to mitigate the effects of ROS. PSII and SQDG proteins both appear reduced in abundance (along with related heat shock proteins) in *C. nivalis* in response to salt, and instead TAGs appear to be favoured. A combination of an increase in acetyl CoA availability, and differences in lipid biosynthesis and catabolism (with *C. reinhardtii* demonstrating early increases in a fatty acid catabolism protein) appears to contribute towards the ability of *C. nivalis* to accumulate fatty acids under salt stress whilst this does not occur in *C. reinhardtii*. The damaging effects of salt on the photosynthetic systems in *C. reinhardtii* appear to interplay with the strain’s salt stress response and lack of lipid accumulation, since PSI suffers greater damage in this strain, whilst *C. nivalis* demonstrates a less damaging reduction in PSII activity instead, protecting itself against salt damage. This was validated by measuring the photosynthesis rates in both species during salt stress, with *C. reinhardtii* demonstrating a much larger decrease in photosynthetic activity than *C. nivalis*.

With the model species *C. reinhardtii* being less halotolerant, more active resources appear to be channelled into maintaining culture health as opposed to going into a resting state and gathering resources for when conditions are more favourable, as happens with the snow alga species, *C. nivalis*, which downregulates the majority of the proteins.

Here, we show how salt acts as a lipid trigger in *C. nivalis. C. nivalis* offers the advantage of robustness to environmental changes and low temperature requirements for bioprocessing. Quantitative proteomic analysis enables elucidation of the mechanisms that lead to increases in fatty acids; this is desirable for maximising lipid production, and therefore for bio-products generation, such as biofuels.

## Materials and methods

### Species selection

Algal species *Chlamydomonas reinhardtii* strain CC-4325 sta1-1 mt- [Ball I7] was obtained from the Chlamydomonas Resource Centre (www.chlamycollection.org). The Ball I7 strain is deficient in the catalytic (small) subunit of ADP-glucose pyrophosphorylase. The strain was derived by X-ray mutagenesis of the wild-type strain CC-4323. This strain was selected because the study aimed to find optimal conditions for potential fatty acid yield of *C. reinhardtii* under salt stress as a comparison to *C. nivalis*. Low starch mutants have historically shown good capacity for lipid and fatty acid yield under nitrogen deprivation, demonstrating that the strain is capable of significant fatty acid accumulation.

Snow algal species *Chlamydomonas nivalis* (strain number CCAP 11/128) was obtained from the Culture Collection of Algae and Protozoa, Scottish Marine Institute, Oban. This strain was originally collected from rocks below a snowfield near Saddlebag Lake, Sierra Nevada, USA.

### Culturing conditions

The media and culture conditions were selected according to the optimal growth requirements of the two species: as neither species is able to grow in the other’s required growth medium [20], two different media and growth setups were used, with *C. nivalis* grown photoautotrophically and *C. reinhardtii* grown mixotrophically. In each case the aim was to maximise the potential fatty acid yield from each species, to test the hypothesis of whether salt acts as a lipid trigger in the two strains. *Chlamydomonas reinhardtii* was grown in TAP medium in 24-h light, at 25 °C, at 175 µEm^−2^ s^−1^ in a Sanyo Versatile Environmental Test Chamber Model MLR-351H on shakers at 100 rpm. *C. nivalis* was grown in 3 N-BBM-V medium, at 16 °C in sealed 500-mL conical flasks submerged in a temperature-controlled water bath, in 24 h light at 40 µEm^−2^ s^−1^. Filtered sterile air was bubbled into the cultures at a flow rate of between 61.0 and 71.8 mL min^−1^, using a pump and tubing with Whatman hepa-vent filters (GE Healthcare Life Sciences, Amersham, UK), to provide a carbon dioxide for a carbon source.

Each culture was grown to mid-log phase in control media, centrifuged at 3000 g for 5 min and then re-suspended at optical density (OD) 0.35 (750 nm) in either control media or media with additional 0.2 M NaCl. Each condition was grown in triplicate. It has previously been established that salt levels beyond 0.2 M NaCl are toxic to *C. reinhardtii* [11]. Samples of 50 mL were taken at regular intervals along the growth curve for each proteomic and biochemical analysis sample.

*C. nivalis* and *C. reinhardtii* cultures were subjected to salt stress conditions over a time-course experiment and compared to control conditions for each species' growth (Fig. 1). Proteomic samples were taken at the onset of salt stress (0 h), and compared against samples taken at the onset of carbohydrate accumulation (80 h) and the onset of fatty acid accumulation (168 h) in *C. nivalis,* as determined using anthrone and FAME analysis, respectively. The time-points of 80 and 168 h correspond to early and mid-log phase growth of *C. nivalis*. The equivalent points of the growth cycle (0, 11 and 18 h, respectively) were sampled for *C. reinhardtii* cultures in order to compare proteomic responses of each species at early and mid-log phase.

### Biochemical analysis

OD was used to estimate culture density during culture seeding, and was measured at 750 nm on an Ultraspec 2100 Pro spectrophotometer (Biochrom Ltd, Cambridge, UK), using 1 mL cuvettes, and using deionised water as a blank. Cell counts were undertaken using a Helber Cell Counting chamber.

Dry cell weight (DCW) was used as a normalising measure of culture growth and volume, as well as for normalisation of all FAME, carbohydrate and chlorophyll measurements. Each biochemical sample was washed in 1 × phosphate buffered saline (PBS) buffer and before freezing and DCW calculations. DCW was measured by centrifuging a sample from the culture using a Hermle Z400K centrifuge (Labnet International Inc., New Jersey, USA) at 1200 g for 10 min at 4 °C, and transferring the pellet to a pre-weighed 1.5 mL micro-centrifuge tube. The sample was frozen, first at − 20 °C, then at − 80 °C, and then freeze-dried. The new tube and sample weight was used to obtain the DCW of the culture. The dried sample was used to carry out biochemical analysis of fatty acids, carbohydrates and chlorophyll analysis.

Fatty acids were quantified via solvent extraction and transesterification to fatty acid methyl esters (FAMEs), followed by gas chromatography with flame ionisation detection (GC-FID). This was selected as the most suitable method for analysing lipid production in this study. The method used was that of Hounslow et al. [11]. Briefly, from a 10 mg mL^−1^ algal suspension, 100 µL sample was added to a 2 mL Eppendorf tube and 500-µL glass beads (425–600 µm, from Sigma (G8772)) were added to the sample, with 1.2 mL of methanol:chloroform (1:2) solution. Samples were disrupted using a Disruptor Genie bead beater (Scientific Industries, New York, USA, Serial No. D68-10,198 and D48-1014) for 10 cycles of 2 min, with 2 min intervals on ice. Samples were centrifuged at 16,000 g at 4 °C for 5 min and the supernatant was transferred to an Eppendorf tube containing 400 µL chloroform and 400 µL HPLC-grade ultrapure water. Samples were centrifuged for 15 min at 7000 g at 4 °C, and the bottom organic layer was transferred to a glass vial and evaporated under inert nitrogen gas. 100 µL 10% BF3:methanol solution and 250 µL chloroform:methanol (1:1) solution were added to each sample and incubated at 80 °C for 90 min, then cooled at room temperature for 10 min.

600 µL hexane and 300 µL ultrapure water were added and samples were transferred to an Eppendorf tube, mixed for 1 min and centrifuged at 7000 g for 5 min at 4 °C. The top organic layer was transferred to a glass vial, evaporated under inert nitrogen gas and resuspended in 100 µL hexane. GC-FID analysis was carried out using a Thermo Finnigan TRACE 1310 Gas Chromatograph with FID detector and autosampler (Thermo Scientific, Hertfordshire, UK), and TRACE TR-FAME capillary column (dimensions 25 m × 0.32 mm × 0.25 µm), calibrated using Supelco 37 Component FAME mix (Supelco, Bellefonte, PA). The data contained three analytical replicates and three biological replicates.

Carbohydrates were measured using a modified version of the anthrone method, as described by Longworth et al. [12]: from a 0.5 µg µL^−1^ algal suspension, 200 µL was added to a glass test tube, followed by adding 400 μL of 75% H_2_SO_4_ and 800 μL of anthrone solution (25 mg anthrone, 500 μL EtOH, 12 mL 75% H_2_SO_4_). Samples were vortexed and then heated to 100 °C for 15 min. Once cooled, samples were vortexed and the OD was read at 620 nm. Carbohydrate concentrations were calculated via a standard curve using known glucose concentrations. This measurement was used because the majority of the carbohydrates in *Chlamydomonas* species are starch [31].

A method based on Wellburn [77] was used for chlorophyll analysis, and was carried out as described in Longworth et al. [12]: from a 10 µg µL^−1^ algal suspension, a 50 µL aliquot was centrifuged to obtain a pellet, which was resuspended in 200 µL phosphate buffer (H_3_PO_4_, pH 7.4, 0.05 M), and 100 µL glass beads (425–600 µm) from Sigma (G8772) were added. Samples were agitated for 10 min in the dark using a Disruptor Genie bead beater. 800 µL acetone was added to each sample, before being incubated in the dark for 10 min, then centrifuged at 1000 g for 1 min. 110 µL of the resulting solution was added to a 400 µL quartz cuvette and the OD was read at 663, 646 and 470 nm (using 80% acetone as a reference). Absorbances were used to calculate chlorophyll content as described in Wellburn [77]. Three biological replicates were used for each phenotype.

### Proteomic sampling and iTRAQ labelling

Algal samples were taken from the cultures, centrifuged at 3000 g and washed in a sucrose-based wash buffer (50 mM Tris, pH 7.5; 100 mM EDTA, pH 8.0) with sucrose adjusted to the salt concentration of the culture to create an isotonic solution (values found in Weast [78]): for 0.2 M NaCl samples, 0.335 M sucrose was added. The samples were centrifuged and stored at -80 °C.

At the time of extraction, lysis buffer 0.5 M tetraethylammonium bicarbonate (TEAB) buffer, with 1% plant protease inhibitor cocktail (Sigma-Aldrich, Poole, UK, catalog no: P9599), was added to the pellet to re-suspend it. The pellet was then ground three times in a pre-chilled mortar and pestle in liquid nitrogen for 15 min each time, before collecting the sample in a 1.5 mL Lo-Bind Eppendorf tube. Samples were sonicated in an ice bath for 5 min. A probe sonicator was used to sonicate for two cycles (a cycle being a single burst) using a Micro tip Branson sonifier (Emerson, Danbury, CT). The samples were centrifuged at 18,000 g for 30 min at 4 °C to separate the insoluble pellet from the soluble protein fraction.

One part extracted soluble protein was added to 4 parts ice-cold acetone (pre-chilled to – 20 °C) and kept at – 20 °C for 12 h. Samples were centrifuged at 21,000 g at 4 °C for 30 min, the acetone was removed and the precipitated protein samples were reconstituted in 0.5 M TEAB. Bradford Ultra Detergent Compatible Coomassie-based protein quantification method was used to measure protein concentration in the samples. 100 µg of sample was suspended in 20 µL of 0.5 M TEAB. Reduction was performed by adding 2 µL 50 mM Tris-(2-carboxyethyl)-phosphine (TCEP) and incubated for 1 h at 60 °C. Samples were alkylated by adding 1 µL 200 mM methyl methane-thiosulfonate (MMTS) and incubating for 10 min at room temperature (20–25 °C) in the dark. A solution of 0.5 mg mL^−1^ trypsin in TEAB was prepared, and a ratio of 1:20 (µg µg^−1^) trypsin to protein was added to the sample. The samples were incubated for 16 h at 37 °C to digest the proteins into peptides. Digested samples were dried down in a Scanvac vacuum concentrator (Labogene, Denmark) and reconstituted in 30 µL 1 M TEAB, pH 8.5. The iTRAQ 8 plex kits were obtained from ABSciex (Warrington, UK), and labelling was carried out as described in the manufacturer's protocol. iTRAQ labels 113 and 114 were used for the 0 h salt stress cultures, labels 115 and 116 were used for the early log time point in salt-stressed cultures (11 and 80 h for *C. reinhardtii* and *C. nivalis*, respectively), labels 117 and 118 were used for mid-log control cultures, and labels 119 and 121 were used for the final salt stress time point (18 and 168 h for *C. reinhardtii* and *C. nivalis*, respectively), with two biological replicates being used in a four-way experimental comparison, as described by Pandhal et al. [79], Ow et al. [26] and Raut et al. [80]. All iTRAQ-labelled samples were then pooled before being dried in a vacuum concentrator.

### Fractionation via Hypercarb column HPLC

Separation of iTRAQ-labelled peptides was carried out on a Dionex UltiMate 3000 Autosampler linked to Dionex UltiMate 3000 Flow Manager and Pump system (Thermo Scientific, Hemel Hempstead, UK). Wash Buffer C was 20% ACN. Samples were re-suspended in 120 µL Buffer A (3% ACN, 0.1% TFA) and loaded onto a Hypercarb™ Porous Graphitic Carbon LC reversed phase Analytical Column with 3 µm particle size, 50 mm length, 2.1 mm diameter and 250 Å pore size (Thermo Scientific, Hemel Hempstead, UK). Buffer A was exchanged with Buffer B (97% ACN, 0.1% TFA) with a flow rate of 0.2 mL min^−1^ with the following gradient: 2% B (0–15 min), 2–30% B (15–80 min), 30–60% B (80–130 min), 60–90% B (130–131 min), 90% B (131–136 min), 2% B (137–145 min). The fractions were collected every two minutes from 20 to 120 min, then dried for 20 h on a Scanvac vacuum centrifuge (Labogene, Allerød, Denmark) connected to a Vacuubrand Vacuum Pump (Vacuubrand, Wertheim, Germany). This separation method has the advantage of not needing de-salting clean up after fractionation. The fractions were then recombined into 6 fractions to run on a LC–MS/MS. The dried fractions were re-suspended in 20 µL Loading Buffer (3% ACN, 0.1% TFA, 96.9% HPLC grade H_2_0), and 5 µL from each fraction was combined in 6 fractions (F) as follows, based on UV intensity (214 nm) and retention time: F1 (minutes 40–46 and 100–106), F2 (minutes 48–56), F3 (minutes 58–66), F4 (minutes 68–74), F5 (minutes 76–86), F6 (minutes 88–98). Samples before 40 min were not injected as these are not peptide rich but consist mainly of excess iTRAQ labelling molecules, which are undesirable for MS sample running. From each of the six pooled fractions, 5 µL was injected into the Q Exactive HF MS.

### LC–MS/MS

Liquid chromatography with tandem mass spectrometry (LC–MS/MS) was performed and analysed by nano-flow liquid chromatography (U3000 RSLCnano, Thermo Scientific, Hemel Hempstead, UK) coupled to a hybrid quadrupole-orbitrap mass spectrometer (QExactive HF, Thermo Scientific, Hemel Hempstead, UK). iTRAQ-peptides were separated on an Easy-Spray C_18_ column (75 µm × 50 cm) using a 2-step gradient from 97% solvent A (0.1% formic acid in HPLC grade H_2_0) to 10% solvent B (0.08% formic acid in 80% acetonitrile) over 5 min then 10% to 50% B over 75 min at 300 nL min^−1^. The mass spectrometer was programmed for data-dependent acquisition with 10 product ion scans (resolution 15,000, automatic gain control 5e4, maximum injection time 20 ms, isolation window 1.2 Th, normalised collision energy 32, intensity threshold 2.5e5) per full MS scan (resolution 60,000, automatic gain control 3e6, maximum injection time 100 ms).

### Data analysis

Six fraction files obtained from each iTRAQ experiment were processed in data analysis software PEAKS7® to enable de novo sequencing, variable post-translational modification (PTM) searching and homology searching. The data for *C. reinhardtii* and *C. nivalis* were each searched against the *C. reinhardtii* UniProt proteome database (taxa id: 3055, downloaded 6 May 2016, 15,172 entries), using the following settings: digestion type: trypsin; variable modifications: oxidation (M); fixed modifications: MMTS; MS scan type: MS^2^; peptide-to-spectrum matches (PSM) FDR 0.01; protein FDR 0.01; site FDR 0.01; labelling: iTRAQ 8-plex; MS tolerance 0.2 Da; MS/MS tolerance 0.2 Da; label mass tolerance 0.01 Da; database: UniProt proteome database for *C. reinhardtii* (taxa id: 3055, downloaded 6 May 2016, 15,172 entries); min peptide length 6; max peptide length 4600; max mis-cleavages 2; maximum charge state 7; min number of unique peptides 1. Data was run through in-house programs to integrate peptide quantification information to the protein level and to perform analysis of variance (ANOVA) based statistical analysis as previously described [81]. A false discovery rate (FDR) of 1% was applied with a requirement of identifying at least two unique peptides per protein identification and *p* < 0.05. The accession numbers of proteins increased and decreased in abundance were searched against the UniProt database (www.uniprot.org) to identify the function of the proteins based on Gene Ontology annotation. KEGG analysis was carried out using the "Search&Color Pathway" tool (see Figures S7 and S8 in Additional file 1).

### Photosynthesis and respiration analysis using oxygen electrode

Photosynthesis and respiration were measured using an oxygen electrode set-up (Hansatech Ltd, Kings Lynn, England) connected to Picolog software (Pico Technology, St Neots, UK). The oxygen electrode set-up was Oxygen Electrode S1 Disc placed into the chamber connected to a L52A light source type, O_2_ Electrode Control Box CB2-D and CO_2_ Light Source L52B Control Box (Hansatech Ltd, Kings Lynn, England). A DrDAQ converter connected the instrument for computer-based control. The instrument was calibrated and voltage measurements of 2-mL samples were taken, according to the method of Smith et al. [82]. The OD of the sample was used to normalise for culture density.

## Supplementary Information

**Additional file 1.** This document contains additional figures, including percentages of individual FAMEs detected in *C. reinhardtii* and *C. nivalis* cultures, PCA plot groupings of iTRAQ labelled *C. reinhardtii* and *C. nivalis* samples, KEGG mapping of proteomic changes to *C. reinhardtii* and *C. nivalis* under salt stress, and preliminary data indicating *C. reinhardtii* and *C. nivalis* growth rates during salt stress.

## Data Availability

The mass spectrometry proteomics data have been deposited to the ProteomeXchange Consortium via the PRIDE [83] partner repository with the dataset identifier PXD018148.
