# Supplementary material for: Quantitative proteomic comparison of salt stress in Chlamydomonas reinhardtii and the snow alga Chlamydomonas nivalis reveals mechanisms for salt-triggered fatty acid accumulation via reallocation of carbon resources
Source: Biotechnol Biofuels. 2021 May 22;14:121. doi: 10.1186/s13068-021-01970-6 (PMC8141184; doi:10.1186/s13068-021-01970-6)
Supplement: Supplementary file 1 — Additional file 1. This document contains additional figures, including percentages of individual FAMEs detected in C. reinhardtii and C. nivalis cultures, PCA plot groupings of iTRAQ labelled C. reinhardtii and C. nivalis samples, KEGG mapping of proteomic changes to C. reinhardtii and C. nivalis under salt stress, and preliminary data indicating C. reinhardtii and C. nivalis growth rates during salt stress. [file 13068_2021_1970_MOESM1_ESM.docx]

# Supplementary Materials


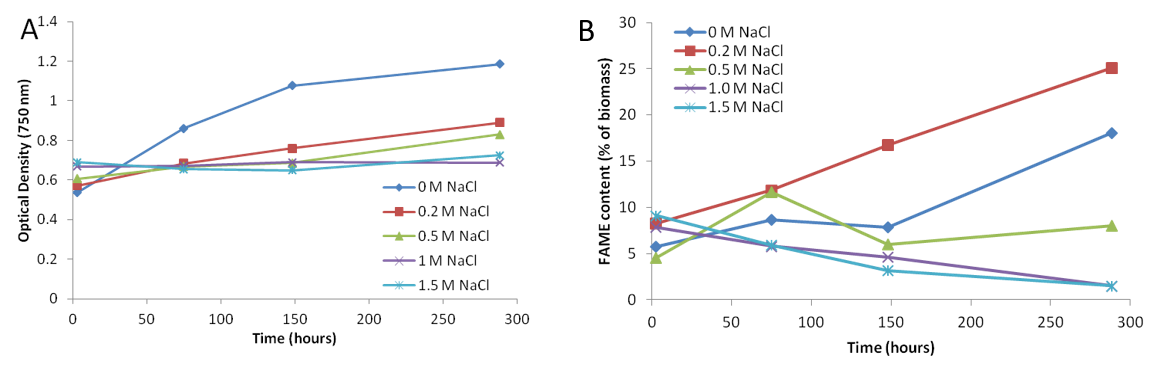


Figure S1 Preliminary pilot data of *C. nivalis* growth.

Growth is represented by OD 750 nm (A) and of FAME content (B), showing a range of salt concentrations used for culturing *C. nivalis* (n=1). 0.2 M NaCl was selected after initial investigation for subsequent experimentation, as the salt concentration was suitable for reducing the growth rate (thereby indicating stress) whilst inducing lipid accumulation. Higher concentrations of salt, whilst also halting growth, were too high and prevented lipid accumulation from occurring.


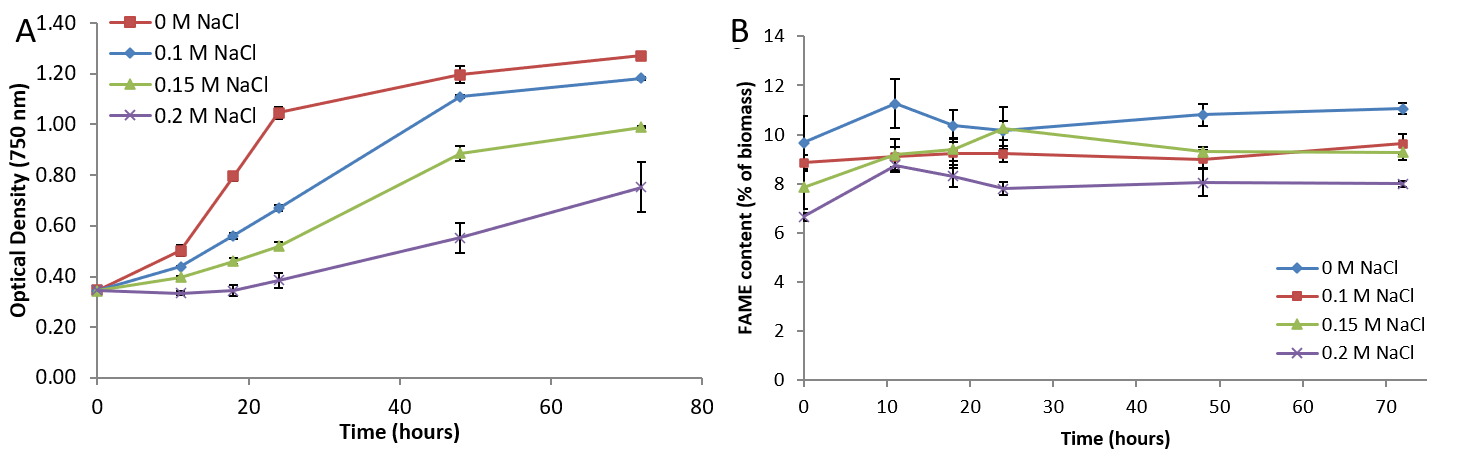


Figure S2 Preliminary data indicating *C. reinhardtii* growth rates.

Growth is represented by OD 750 nm (A) and of FAME content (B) in *C. reinhardtii* under a range of growth regimes (0, 0.1, 0.15 and 0.2 M NaCl) (n=3). Results indicated that 0.1 and 0.15 M NaCl were not sufficiently high enough salinity to halt culture growth, but that 0.2 M NaCl could halt culture growth. None of the salinities induced lipid accumulation. 0.2 M NaCl was selected as a salinity stressor for both *C. reinhardtii* and *C. nivalis* in subsequent experiments since it induced the same effect of arresting growth in both species, whilst not being toxic enough to kill the cultures.


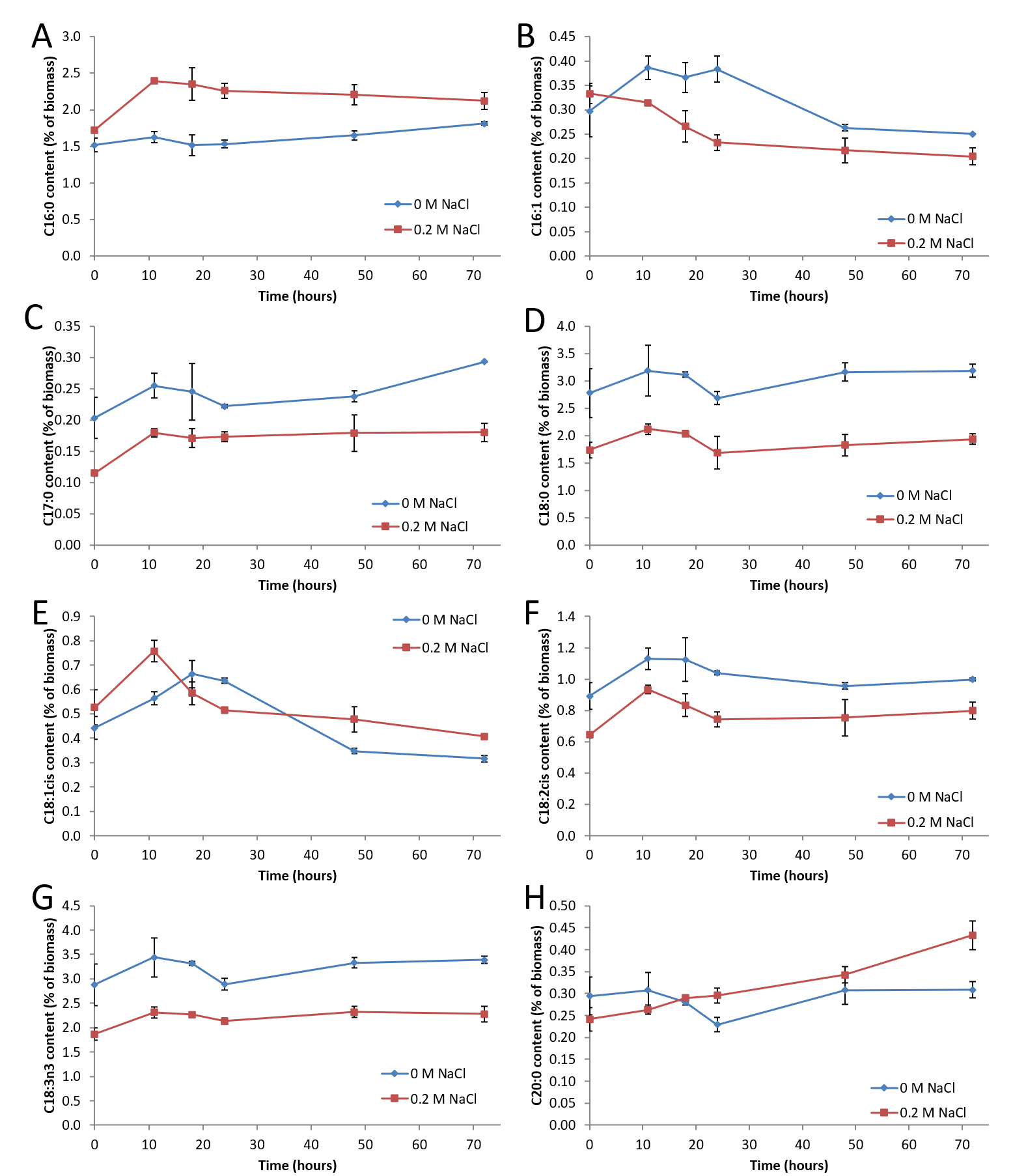


Figure S3 Percentages of individual FAMEs detected in *C. reinhardtii* cultures.

Percentage content of main individual FAME types in algal biomass in *C. reinhardtii* under 0 M (control) and 0.2 M Nacl. FAMEs shown are C16:0 (A), C16:1 (B), C17:0 (C), C18:0 (D), C18:1cis (E), C18:2cis (F), C18:3n3 (G) and C20:0 (H) (n=3).


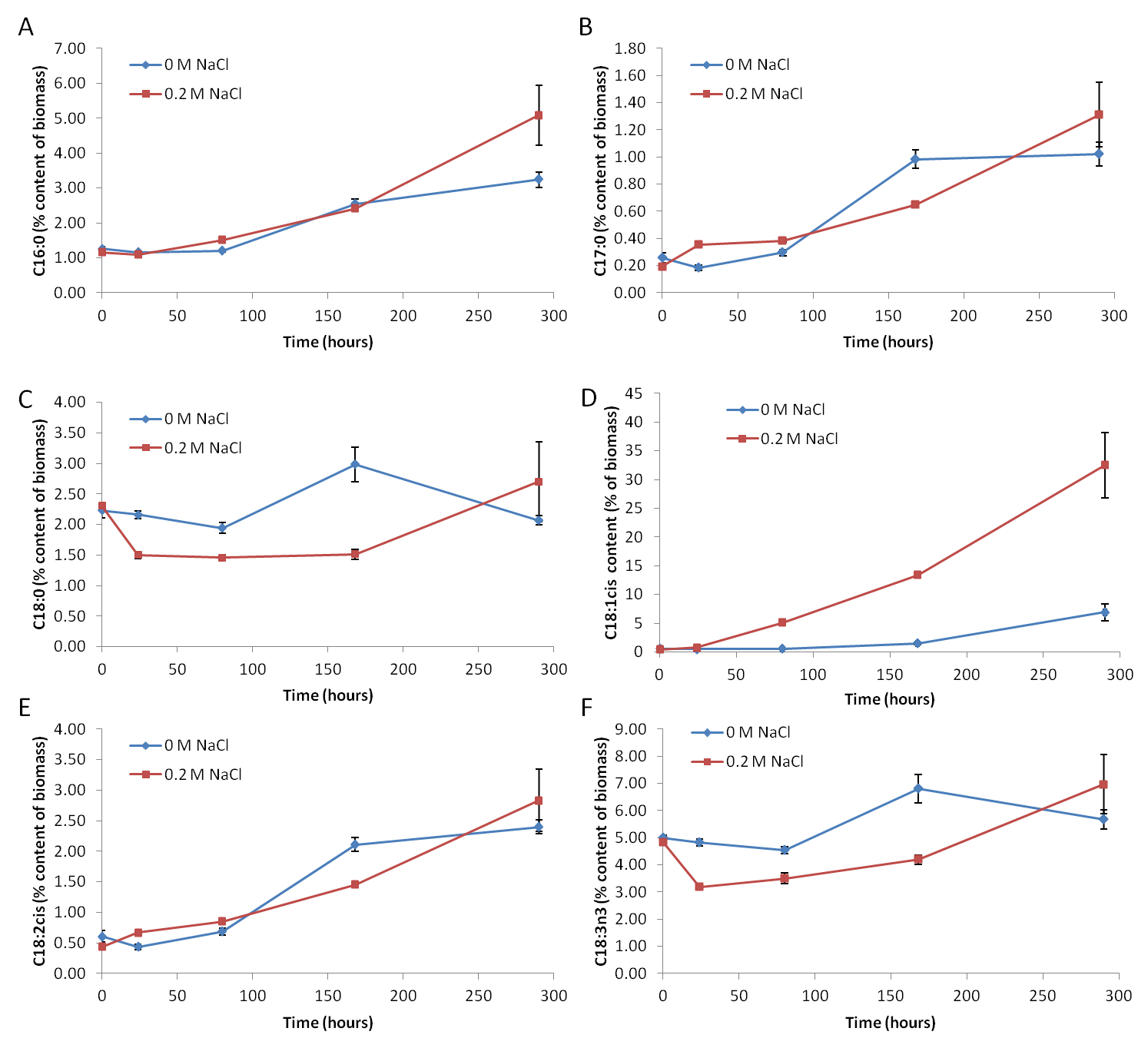


Figure S4 Percentages of individual FAMEs detected in *C. nivalis* cultures.

Percentage content of main individual FAME types in algal biomass in *C. nivalis* under 0 M (control) and 0.2 M Nacl. FAMEs shown are C16:0 (A), C17:0 (B), C18:0 (C), C18:1cis (D), C18:2cis (E), and C18:3n3 (F) (n=3).


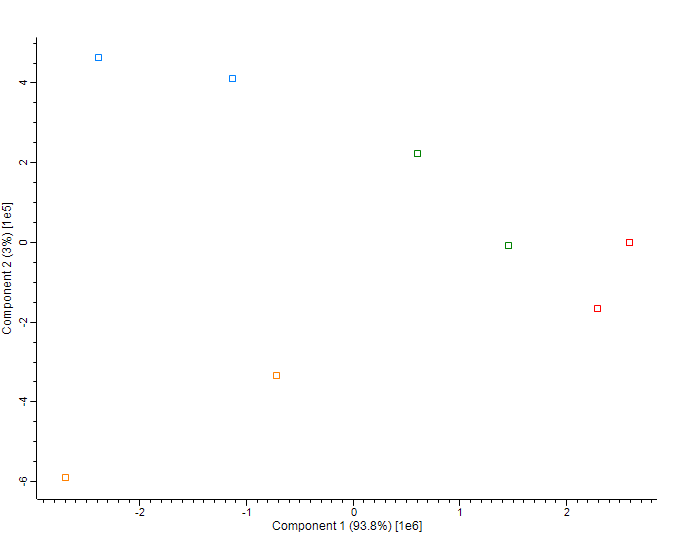


Figure S5 PCA plot groupings of iTRAQ labelled *C. reinhardtii* samples.

Clusters show 0 hour salt conditions (red), 11 hour salt conditions (orange), 18 hour salt conditions (blue) and 18 hour control conditions (green).


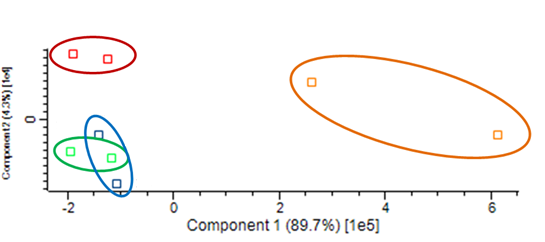


Figure S6 PCA plot groupings of iTRAQ labelled *C. nivalis* samples.

Clusters show 0 hour salt conditions (red), 80 hour salt conditions (blue), 168 hour salt conditions (green) and 168 hour control conditions (orange).


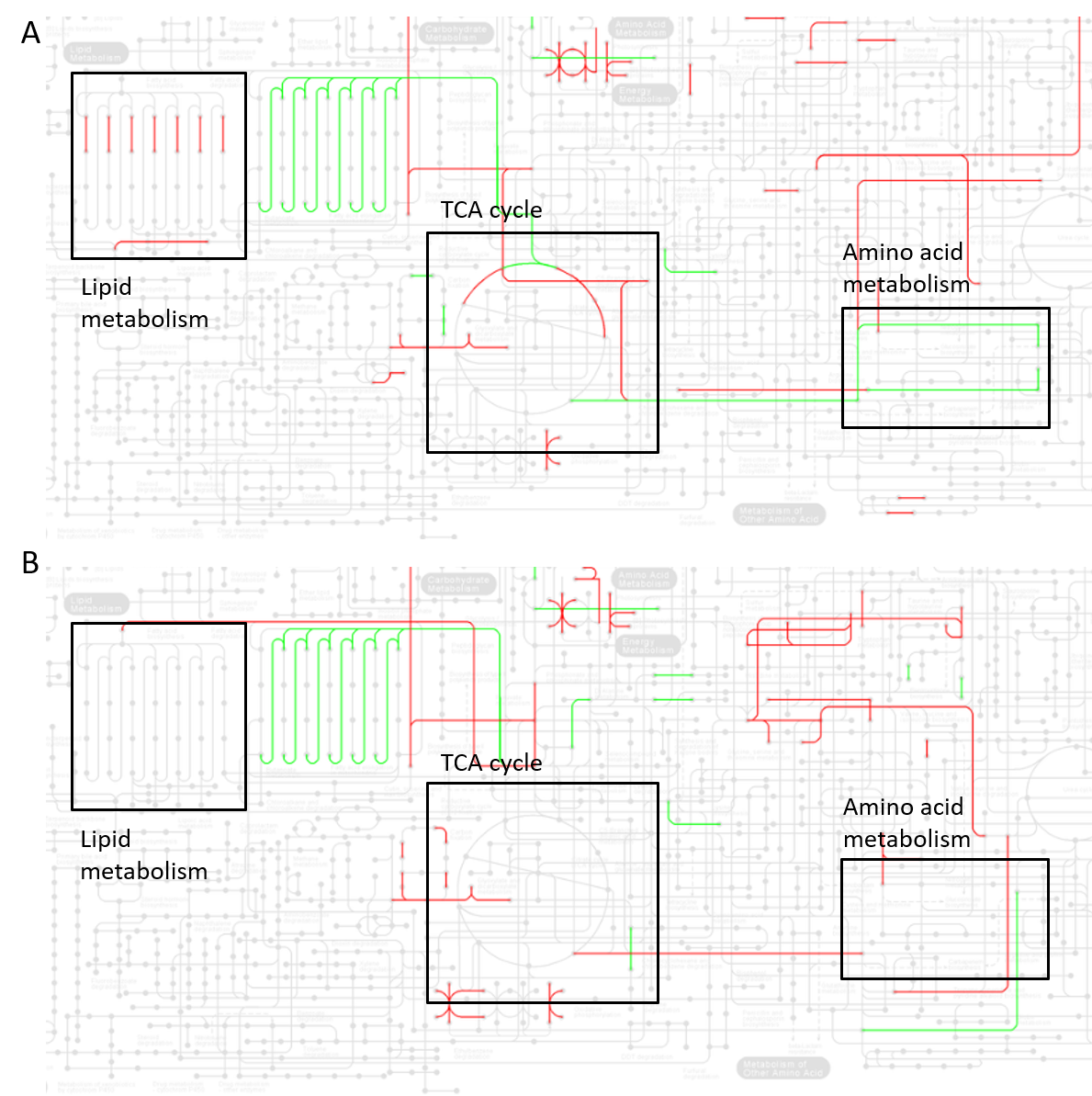


Figure S7 KEGG mapping of *C. reinhardtii* (A) and *C. nivalis* (B) using salt stress time course.

Significant changes are shown for down-regulation (red) and up-regulation (green) between the first and last sample point in salt stress conditions. Highlighted boxes show the differences between the two species in lipid metabolism, TCA cycle and amino acid metabolism.


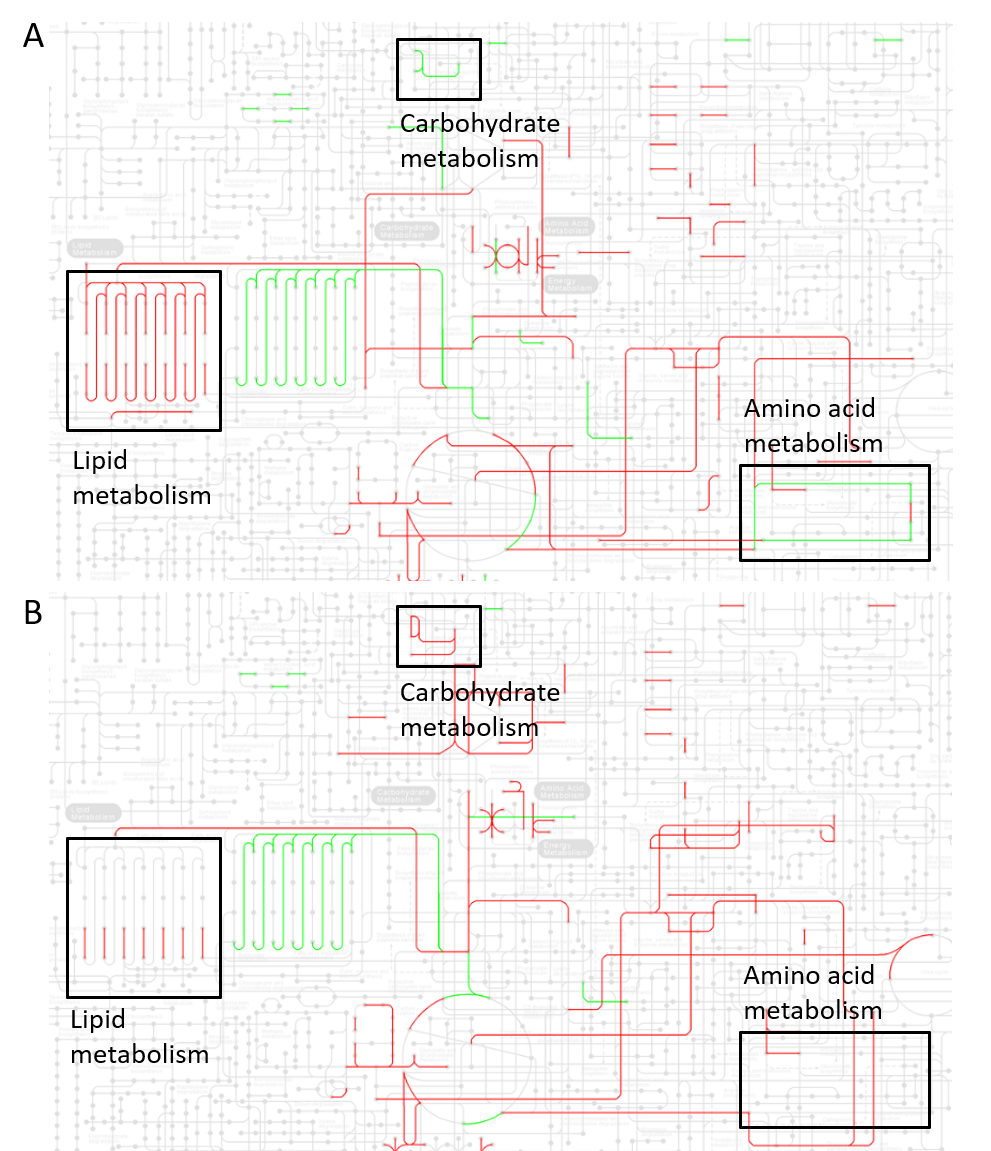


Figure S8 KEGG mapping of *C. reinhardtii* (A) and *C. nivalis* (B) using control versus salt stress.

Significant changes are shown for down-regulation (red) and up-regulation (green) from control conditions to salt stress conditions. Highlighted boxes show the differences between the two species in lipid metabolism, carbohydrate metabolism and amino acid metabolism.
